# Artificial Nuclear Pore Complexes with Exceptionally Selective Shuttle-Cargo Transport

**DOI:** 10.1021/acsnano.6c05720

**Published:** 2026-06-29

**Authors:** Jesper Medin, Bagus Santoso, Leyla Beckerman, Radhika Vattikunta, Rebekah Hailes, John Andersson, Andreas Dahlin

**Affiliations:** Department of Chemistry and Chemical Engineering, 11248 Chalmers University of Technology, Gothenburg 41296, Sweden

**Keywords:** nuclear pore complexes, nanopores, polymer brushes, nucleic acids, poly(hydroxyethyl acrylamide), poly(methacrylic acid), selective transport

## Abstract

The nuclear pore complex (NPC) is a large protein assembly that controls transport of macromolecules to or from the nucleus in eukaryotic cells. It is capable of facilitated transport in which “cargo” species can bind to “shuttles”, which specifically translocate the NPC, while the cargo alone cannot pass. In order to better understand the transport mechanism, attempts have been made to reconstruct the NPC transport using synthetic systems (bottom-up). However, it has proven difficult to achieve a functioning shuttle-cargo transport mechanism, in particular with high selectivity. Here, we present fully artificial NPCs based on heteromolecular polymer complexation. Polymer brushes consisting of poly­(hydroxyethyl acrylamide) are prepared on solid-state nanopores to form a barrier that generally only allows small molecules (a few kg/mol) to pass. Still, at lowered pH, multivalent interactions with poly­(methacrylic acid) enable efficient transport of this polymer through the brush barrier (predicted max rate >1000 molecules per pore per second). By fine-tuning the affinity, which is strongly dependent on factors such as pH and molecular weight, we show that the polymer shuttles can diffuse through the brush barrier without strongly altering its morphology. As a mimic of nucleic acid export through the NPC, we show that DNA cargo strands conjugated to the polymer shuttles translocate the pores, even though they are too large to pass in their free form. We consider the selectivity of our system to be exceptional, since there is no detectable leakage of unconjugated macromolecules, not even in the presence of free transport shuttles. Besides being of fundamental interest to understand soft matter in general and the NPC in particular, the possibility to switch transport on/off with pH enables unique applications of nanopore-based structures. As an example, we show secure, tether-free, and noninvasive trapping of molecules inside nanoscale chambers a few attoliters in volume under physiological conditions.

## Graphical abstract

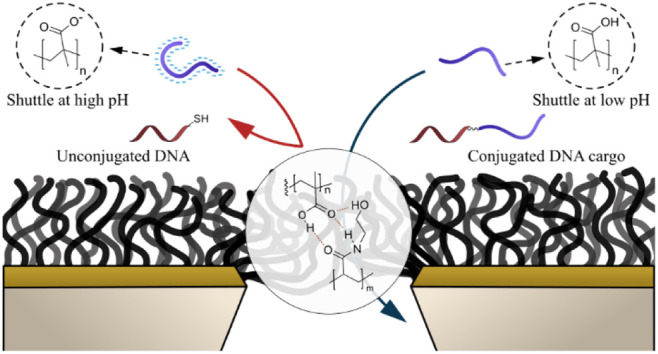

## Introduction

The ability to mimic biological systems by artificial components has improved our understanding of life and led to many new technologies, following the motto “what I cannot create, I do not understand” (due to Richard Feynmann). A remarkable example of a complex nanoscale machinery found inside cells is the nuclear pore complex (NPC). The NPC is a large protein assembly in the nuclear envelope that regulates molecular traffic to and from the cell nucleus by forming a selective barrier that generally restricts the passage of large molecules (40 kg/mol or more). Yet certain proteins, known as karyopherins^1^ are able to interact with the NPC and diffuse through it quickly and passively, in the sense that no chemical net reaction occurs throughout the translocation event.^2^ Furthermore, large molecules can be transported through the NPC if they are bound to karyopherins. This “shuttle-cargo” transport mechanism is particularly interesting because it shows that the NPC interior is not merely a size-selective barrier. The shuttle-cargo complex is larger than the cargo, but still the cargo does not translocate on its own. To achieve this feature, the NPC has evolved a dynamic structure that responds to specific interactions with karyopherins in a complex manner.^3^ Indeed, the transport mechanism and the associated structural changes continue to be a topic investigated by many research groups worldwide, using a great variety of methods.^4^ Focus lies on the behavior of the intrinsically disordered proteins grafted to the NPC interior channel, known as phenylalanine-glycine repeat nucleoporins (FG-Nups). Transport through the NPC is also central in many medical contexts, such as gene therapy.^5^

Several studies have used chemically modified solid-state nanopores to reproduce the transport selectivity of the NPC in an artificial setting, enabling detailed biophysical investigations.^6^ Kowalczyk et al. used nanopores in silicon nitride modified with FG-Nups and resistive pulse sensing to verify the selective transport of karyopherins vs bovine serum albumin (BSA).^7^ More work has followed based on the same approach,^8,9^ and optical detection has also been implemented.^10^ However, an actual shuttle-cargo transport mechanism, i.e., not just selectivity with respect to different proteins, has only been demonstrated in a few cases.^3^ The first ones predate the single-molecule studies: Jovanovic-Talisman et al. used pores in polycarbonate membranes to which FG-Nups were end-grafted.^11^ These artificial NPCs were able to qualitatively reproduce the shuttle-cargo transport mechanism using karyopherins. Caspi et al. used an entirely artificial system based on nanopores modified with poly­(*N*-isopropylacrylamide).^12^ The same polymer was used for the barrier as for the shuttles, and transport of conjugated DNA was demonstrated. More recently, Wang et al. presented a new approach based on coacervates inside pores and demonstrated the transport of DNA strands by zwitterionic polymer shuttles.^13^ However, in all these studies, the selectivity was low: the cargo species, as well as other molecules, were leaking through the pores at rates comparable to those of the facilitated transport. Thus, a pertinent question is whether it is possible to construct an NPC, in a bottom-up manner, with a shuttle-cargo transport mechanism that has good selectivity. To truly mimic the NPC, the shuttle-cargo complex should be selectively transported (over the cargo) even in the presence of free shuttle molecules. This means that the interactions with the shuttle should not simply cause the pores to open for all species. This point appears to never have been explicitly discussed in the literature to date.

In this work, we present artificial NPCs based on multivalent interactions between hydrophilic polymers. By using a hydrophilic neutral polymer brush on solid-state nanopores, we create a strong barrier that does not allow biomacromolecules to pass, while still being able to form intermolecular complexes with poly­(carboxylic acid)­s. Our system enables shuttle-cargo transport with very high selectivity, as demonstrated with DNA as cargo. Furthermore, the system is pH-responsive since the polymer complexation does not occur for ionized chains, which makes it possible to switch the transport on and off. Besides extending our understanding of how transport can occur through “soft nanopores”, we also investigate potential applications based on our NPC mimic. This includes compatibility with click-chemistry for attaching cargo molecules and the possibility to use the transport for tether-free confinement of biomolecules inside nanoscale volumes.

## Results and Discussion

The principle of our artificial NPC and the transport mechanism based on pH-responsive polymer interactions is outlined in Figure 1. The pore diameter was kept in the range 100–150 nm in this study. Although we could also make the pores small enough (∼40 nm) to match the NPC inner diameter,^14^ our focus was here on reproducing the shuttle-cargo transport system rather than size-matching. To create a selective barrier, we utilized the well-known ability of polymers containing carboxylic acid groups to form complexes with other hydrophilic polymers when in their protonated state, an effect typically attributed to hydrogen bonds.^15^ Previously, we have shown that these interactions also occur when one polymer is in the brush configuration, i.e., consisting of end-grafted chains with a surface coverage high enough to promote stretching into the liquid environment.^16,17^ For instance, if poly­(methacrylic acid) (PMAA) is added in the solution phase to a poly­(ethylene glycol) (PEG) brush, this strongly alters the brush properties if the pH is low enough for the heteromolecular interactions to occur.^18^ Although PEG brushes are excellent barriers toward proteins,^19^ we here aimed to investigate a broader variety of brushes and to make them thick enough to securely seal also relatively large (>100 nm) nanopores. Hence, we used atom transfer radical polymerization^16^ (ATRP) with neutral and hydrophilic monomers^17^ to identify a suitable brush with antifouling barrier properties. At the same time, this polymer brush should also exhibit multivalent binding with PMAA, our tentative shuttle molecule.

**Figure 1:**
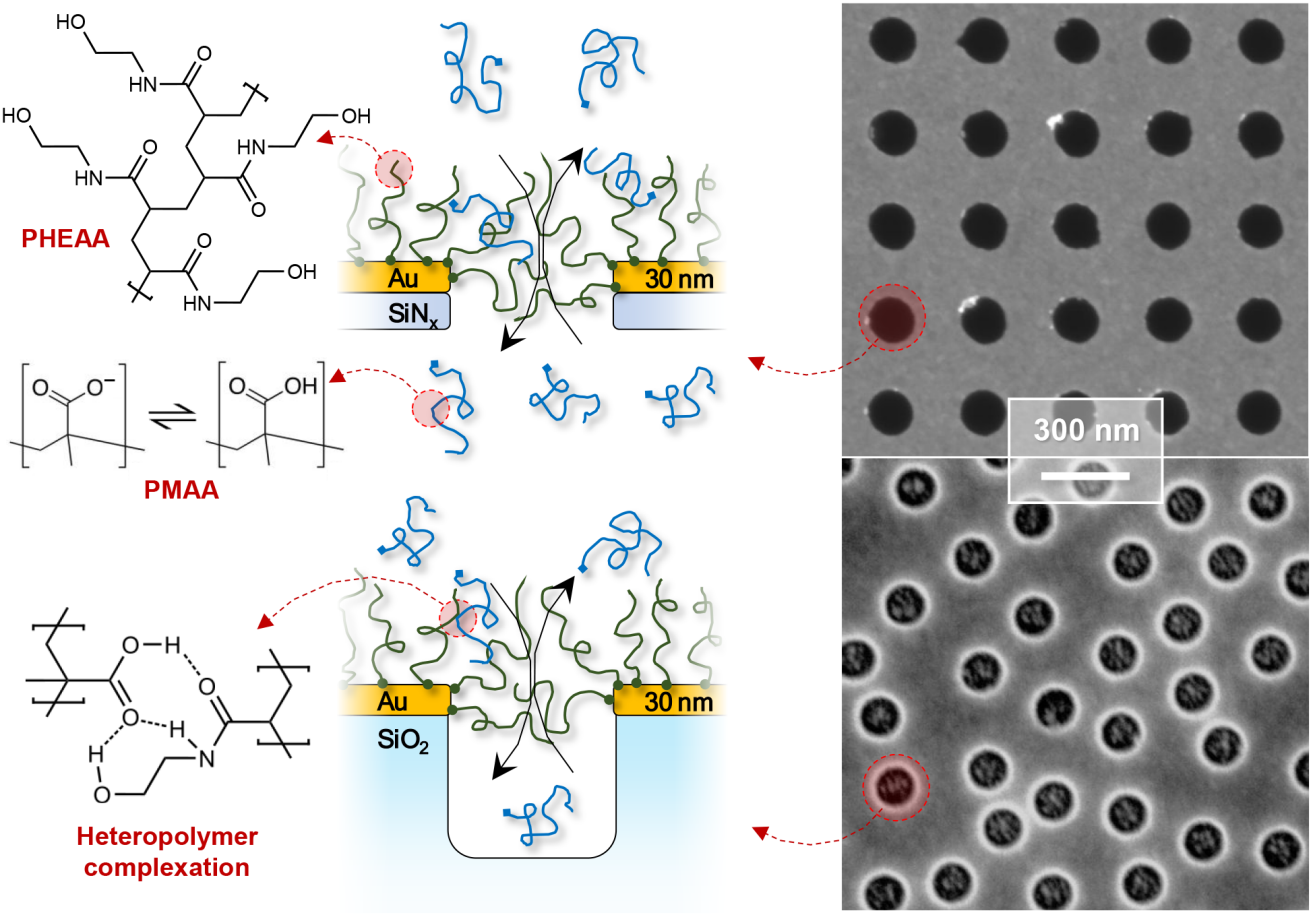
System design. The NPC is mimicked using chemically modified solid-state nanopores. At low pH, poly­(methacrylic acid) becomes neutralized and interacts with poly­(hydroxyethyl acrylamide). This makes the polymers in solution able to diffuse through a nanopore with a brush barrier, potentially even when conjugated to other molecules, which would otherwise not pass. At physiological pH, the carboxylic acid groups are mostly ionized, and the interaction does not occur. The electron microscopy images show the two types of nanostructures used in the study (same scale), one being nanopores connecting two reservoirs and the other “nanochambers” consisting of small cavities in silica. The polymer brush is prepared selectively on gold and seals the apertures in both cases. Note that the surface density of polymers is reduced in the schematics for clarity.

We found that excellent antifouling performance was achieved with poly­(hydroxyethyl acrylamide) (PHEAA) brushes, in agreement with previous reports.^20,21^ The growth of PHEAA by ATRP and the antifouling performance were quantified by surface plasmon resonance (SPR). The thickness for different polymerization times was obtained from SPR spectra measured in the dry state (Figure 2A) using Fresnel modeling.^22–25^ The linear kinetics (Figure 2B) illustrate controlled growth and no significant rate of termination events. The swelling of the polymer brushes was characterized by the noninteracting probe method,^18,23–26^ which provides an “exclusion height”, corresponding to the distance from the surface at which a macromolecule (here 35 kg/mol PEG) cannot penetrate further into the brush (see data in Figure S1). Based on the dry thicknesses and exclusion heights, the PHEAA brushes had a solvent content of about 70%, independent of thickness. It should be noted that this value represents an average degree of hydration inside the brush. On a planar surface, the polymer volume fraction will follow a parabolic function with distance from the surface under good solvent conditions, based on theory^27^ and experiments.^28^ In a pore geometry, the density profile is expected to be more uniform,^29^ although for our short pores, the opening also comes into play, and chains are likely to stretch out into the nearby reservoir.^30^

**Figure 2:**
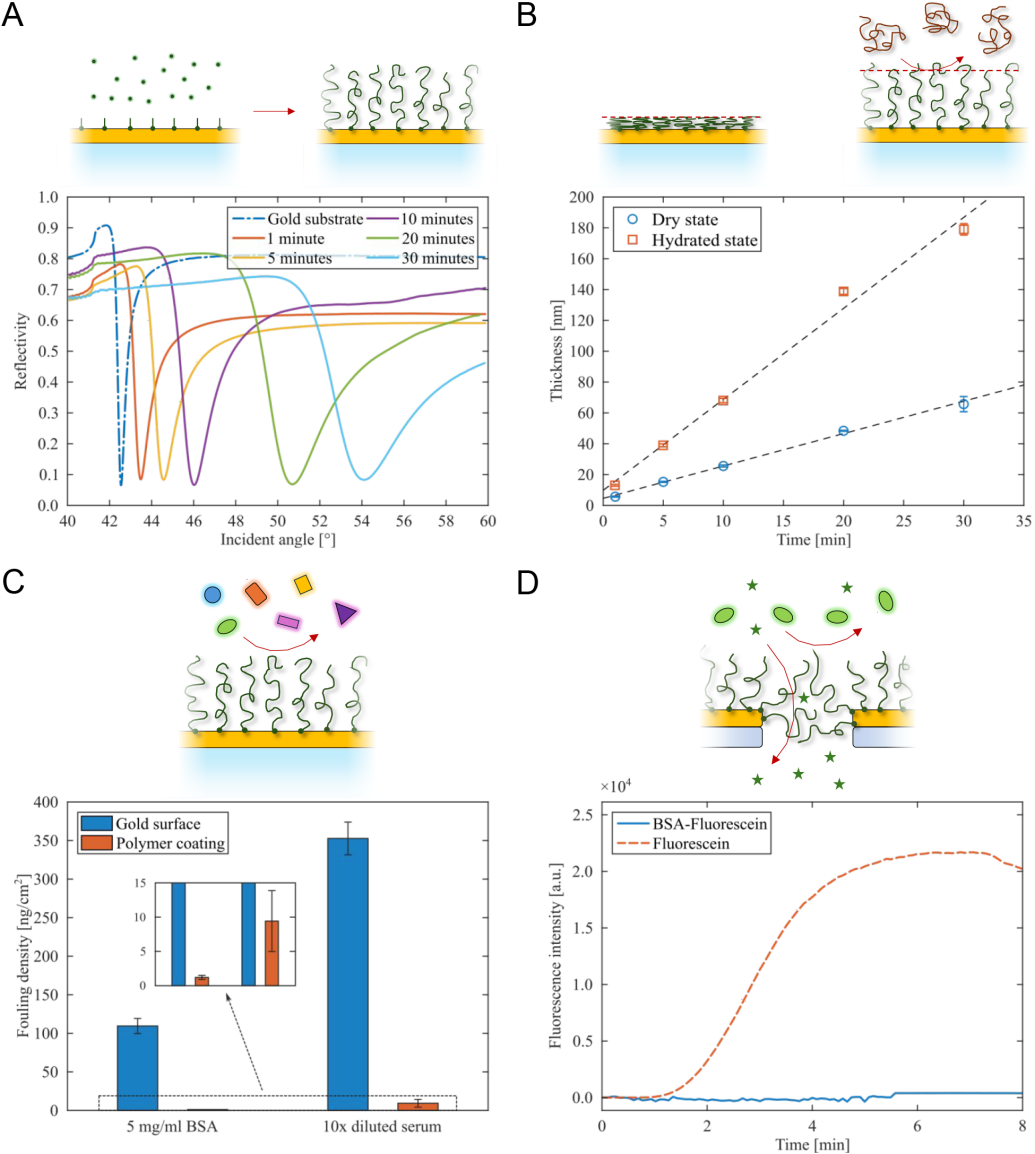
ATRP kinetics and antifouling performance. (A) SPR spectra (670 nm) in air of poly­(hydroxyethyl acrylamide) samples for different polymerization times. (B) Thickness values for dry and hydrated states. The lines are fits to the data points. Error bars represent 95% confidence intervals based on different surfaces and SPR wavelengths. (C) Quantified fouling density for bare and brush-modified gold after exposure to BSA (5 g/L) or 10× diluted serum for 20 min. Error bars show two standard deviations. (D) Exposing PHEAA-modified pores to fluorescein-labeled BSA (injected at 20 μg/mL). No significant fluorescence is detected at the other side of the membrane, while free fluorescein (5 μM) gives a clear signal.

The antifouling performance of PHEAA was quantified by exposing the surfaces to BSA and serum while monitoring the SPR signal (example in Figure S2). For brushes with dry thickness of ∼30 nm, the adsorbed protein amount from serum was reduced by almost 99%, down to 4 ng/cm^2^ at best (Figure 2C). This is considered to be ultralow fouling and on par with optimized brushes used for SPR sensing in complex media.^31^ Interestingly, the amide group in PHEAA seemed to contribute significantly to the antifouling properties, since poly­(hydroxyethyl acrylate), where the nitrogen is replaced by oxygen, exhibited weaker antifouling capacity (data not shown), in agreement with previous work^21^. Based on these results, we expected the PHEAA brushes to form a strong barrier against biomolecules when prepared on nanopores, given that their thickness is comparable to or higher than the pore radius.^19,23^ To confirm this, we monitored the (lack of) transport of fluorescently labeled BSA through PHEAA-modified nanopores (Figure 2D). At the same time, the free dye (fluorescein, 376 g/mol) could diffuse easily through the brush barrier, as expected.^19,23^ This also confirms the wetting of the pores and that liquid exchange occurs across the nanopores through the brush barrier.

Next, we characterized the presumed multivalent complexation between PMAA and PHEAA and its pH sensitivity. Throughout this study, we used PMAA with an average molecular weight of 5 kg/mol (∼58 monomers) unless otherwise stated. Quartz crystal microbalance with dissipation monitoring (QCMD) was used to identify the critical pH at which the heteromolecular polymer complexation started. Indeed, as the pH and degree of ionization for PMAA decreased, binding started to occur to the hydrated brush (Figure 3A). When rinsing with physiological pH buffer, all bound PMAA was always released very quickly. The complexation is expected to cause large changes in hydration, for which the QCMD signals are very sensitive.^18^ Looking at the equilibrium signals for PMAA at different pH, we observed a nonlinear increase in the frequency response vs pH (Figure 3B). Interestingly, around pH 4, the dissipation signal peaked and then turned negative, at all overtones monitored. An increase in dissipation is likely caused by bound PMAA chains that do not fully penetrate into the brush, while a decrease strongly suggests that PMAA has moved into the brush and caused it to collapse due to the multivalent interactions.^18^ Such behavior has been described theoretically by Coalson and coworkers.^29,32^ Hence, we argue that in order to mimic the NPC barrier, neither very weak nor very strong interactions are beneficial. If the PMAA cannot move deep into the brush, it will probably not pass through the brush-modified nanopores. On the other hand, if the interaction is so strong that the brush morphology changes, the pores may “open up” entirely and let all species pass, thereby losing selectivity. Indeed, such behavior has been observed both in experiments^23,33^ and simulations.^29,32^ Hence, we concluded that pH 4, where the *f* and *D* signals peaked, most likely corresponds to a balanced situation where PMAA binds reasonably strongly to PHEAA while it is also able to move within the brush without collapsing it. To the best of our knowledge, complexation with poly­(carboxylic acid)­s has not yet been reported for PHEAA, but the effect is not surprising as it occurs for a great variety of similar hydrophilic polymers, including the structurally similar poly­(hydroxyethyl acrylate).^15^ Interestingly, we also found that the affinity between the polymers increased with temperature (Figure S3), strongly suggesting that hydrophobic interactions also come into play in addition to the presumed hydrogen bonds.^15^ Considering the molecular structures, it seems likely that the side groups interact by hydrogen bonding, while the backbones exhibit hydrophobic interactions.

**Figure 3:**
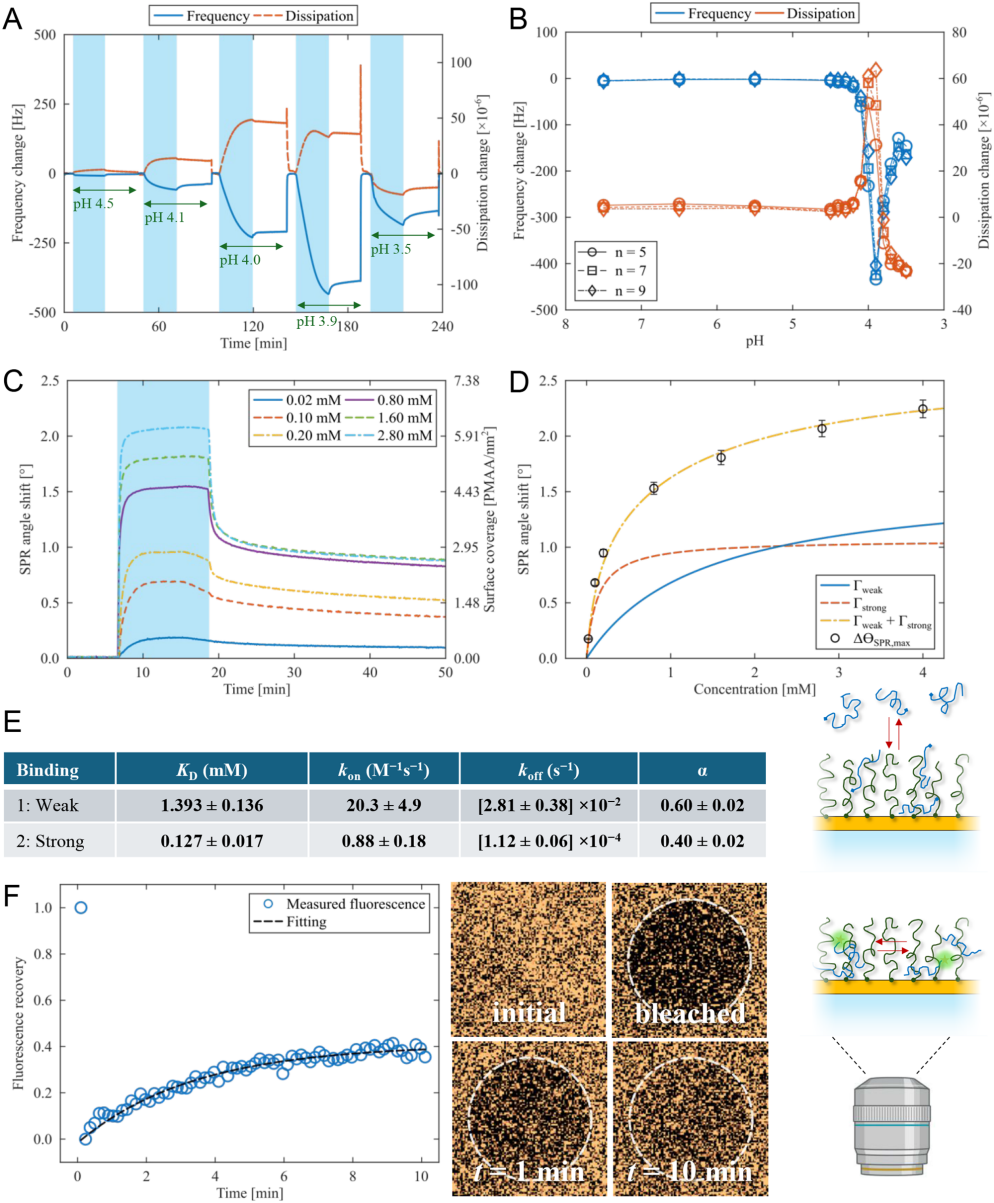
Characterization of multivalent complexation between the two different polymers. (A) Example of QCMD signals during injections of 5 kg/mol PMAA (1 mM) to a PHEAA brush at different pH. After each injection, the system is first rinsed without changing pH and then rinsed by pH 7.4 buffer. (B) QCMD signals at different overtones when in equilibrium with 1 mM PMAA, as a function of pH. Note that the dissipation signals eventually become negative. (C) SPR kinetics of PMAA binding and release at different concentrations (dry PHEAA thickness ∼60 nm). The bulk response has been corrected for.^34^ (D) Dual Langmuir isotherm model fit to the data. Error bars represent two standard deviations obtained from repeated injections. (E) Table of parameters obtained from analysis of affinity and kinetics. The dissociation constants were determined from dual exponential decay fits to the dissociation curves. *K*_D_ values and α were determined by fitting to two independent Langmuir equilibria. The association rate constants were then obtained as *k*_on_ = *k*_off_/*K*_D_. (F) Fluorescence recovery after photobleaching a planar surface PHEAA brush with strongly bound PMAA. Imaging was done by confocal scanning (through 30 nm gold). The images show the intensity distribution at the bleached spot.

Further characterization of PMAA binding in terms of kinetics and affinity was done by SPR due to the ease of translating the response into surface coverage Γ. Figure 3C shows injections of PMAA at different concentrations to PHEAA brushes at pH 4.0. The SPR signal is clearly much larger than that corresponding to a monolayer of PMAA,^18^ which means that the polymers must be binding in multilayers inside the brush. Furthermore, the association is clearly fast, establishing equilibrium in ∼1 min at concentrations above 0.1 mM, while the dissociation showed a partially fast phase where around half of the PMAA left the surface in ∼1 min, followed by a much slower dissociation rate. Overnight monitoring confirmed that the slow release continued for 24 h (Figure S4), which made us to conclude that all PMAA molecules were reversibly bound, but with different affinities. Furthermore, dissociation curves could not be fitted to a single exponential decay, but followed a double exponential function well (Figure S5). Thus, we used a dual-mode Langmuir isotherm with two independent types of binding sites^35^ to describe the interaction:

1 $$\frac{\Gamma }{\Gamma_{\max}}=\alpha \frac{C_{0}}{C_{0}+K_{D1}}+\left[1-\alpha \right]\frac{C_{0}}{C_{0}+K_{D2}}$$

Here, *K*_D1_ and *K*_D2_ are the dissociation constants of the “weak” and “strong” binding states, respectively, while α is the fraction of binding sites that are weak. Note that the ratio Γ/Γ_max_ is proportional to the SPR angular shift,^33^ which enables simple analysis of the data. We include the corresponding surface coverages in Figure 3C based on the refractometric increment of PMAA (see Figure S6 and associated discussion). It is clear from the highest signals in Figure 3C that the saturated surface coverage, in terms of monomers per area of PMAA is comparable to that of PHEAA, suggesting a 1:1 stoichiometry for the monomers, as observed in other polymer complexes.^15^ The equilibrium signals for strong binding were extracted by assuming that, after 6 min of dissociation, all weakly bound PMAA had left, while practically all strongly bound PMAA remained, in agreement with the determined *k*_off_ values. The equilibrium signals for weak binding were then obtained as the difference between plateau values and strong binding signals. Next, the individual *K*_D_ values and α were determined by fitting to separate Langmuir equilibria and combined to the dual binding model (Figure 3D). All values are summarized in Figure 3E. It is noteworthy that the weak binders exhibit very high values for both association and dissociation rate constants, suggesting efficient passage through PHEAA-modified nanopores. We emphasize that all values determined are specific for pH 4.0 and a molecular weight of 5 kg/mol for PMAA. Besides pH, other values for molecular weight,^18^ or even ionic strength^36^ will change the interaction affinity drastically. The brush thickness could, in principle, also influence the *k*_on_ and *k*_off_ values (not investigated in detail), but this is considered less likely since the degree of hydration of the brush is unaltered with thickness (Figure 2B).

Remarkably, exactly the same kind of dual binding model (eq 1) has been used to describe karyopherins interacting with brushes made of FG-Nups.^35^ Thus, our entirely artificial system seems to resemble the real NPC in unexpected ways. Indeed, one theory of the transport mechanism to the nucleus is that some karyopherins are strongly bound to FG-Nups, potentially contributing to upholding the barrier function, while others are more loosely bound with the purpose to enable fast transport.^37^ In our system, the “strong” binding sites could, in principle, involve interactions with the underlying surface^18^ (here, the ATRP initiator layer). However, additional SPR experiments without PHEAA showed that, although PMAA did interact to some extent with the bare initiator layer (Figure S7), the signals were too small to correspond to those from strong binding to the brush. Furthermore, fluorescence recovery after photobleaching (FRAP) showed that even the strongly bound PMAA exhibited mobility inside the brush (Figure 3F). The lateral diffusivity was found to be 0.32 ± 0.09 μm^2^/s, which is comparable to receptors in cell membranes. We note that it is possible to implement more advanced models that account for transitions between weak and strong binding states.^33^ However, we consider this unnecessary since the data were so well described by independent binding to weak and strong sites.

To verify that the PMAA shuttles could be transported through nanopores modified with PHEAA brushes, we first used “nanochambers” consisting of ∼1 attoliter cavities in silica.^23^ The cavity volume is accessed through an aperture in the gold layer on top which is structurally identical to the pores in the silicon nitride membranes.^38^ The exclusion height of PHEAA was kept at ∼100 nm to seal the apertures. We further labeled the polymers in solution by conjugating a dye to a 5 kg/mol PMAA batch with a terminal amine group. This extra chemical group had negligible influence on the interaction with PHEAA (Figure S8). The nanochambers were imaged by epi-fluorescence microscopy (Figure 4A) and a planar gold region on the surface was used as a control.^23^ We hypothesized that the pH-sensitive nature of the polymer interactions should make it possible to trap PMAA molecules inside the nanochambers. The fluorescent PMAA was “lured” into the traps at low pH, after which the pH was increased to physiological, making the PHEAA barrier impenetrable. Given that the concentration of PMAA in the reservoir during trapping is comparable to that corresponding to one molecule inside a nanochamber (∼1 μM), it is expected that one or a few molecules will be left inside each nanochamber when the polymer interactions cease. We incubated the PHEAA nanochambers with 50 μM 50% labeled PMAA at pH 4.0 for 30 min, after which molecules in solution were rinsed away and the pH increased to 7.4. Indeed, clear fluorescence signals from the nanochambers were then observed (Figure 4A). As a control, if the pH was never lowered to induce the interactions, there was no fluorescence signal after rinsing. Similarly, when performing the same experiment on nanochambers with PHEAA brushes that were too short to seal the apertures, i.e., a brush thickness significantly lower than the radius, there was again no remaining fluorescence signal after going back to pH 7.4. This also verifies that there was no adsorption of PMAA inside the nanochambers, as expected since the polymer has the same charge as the silica interior surface. The PMAA remained securely trapped in the chambers for at least a time scale of hours. (Precise measurements of such long trapping times are difficult due to bleaching.)

**Figure 4:**
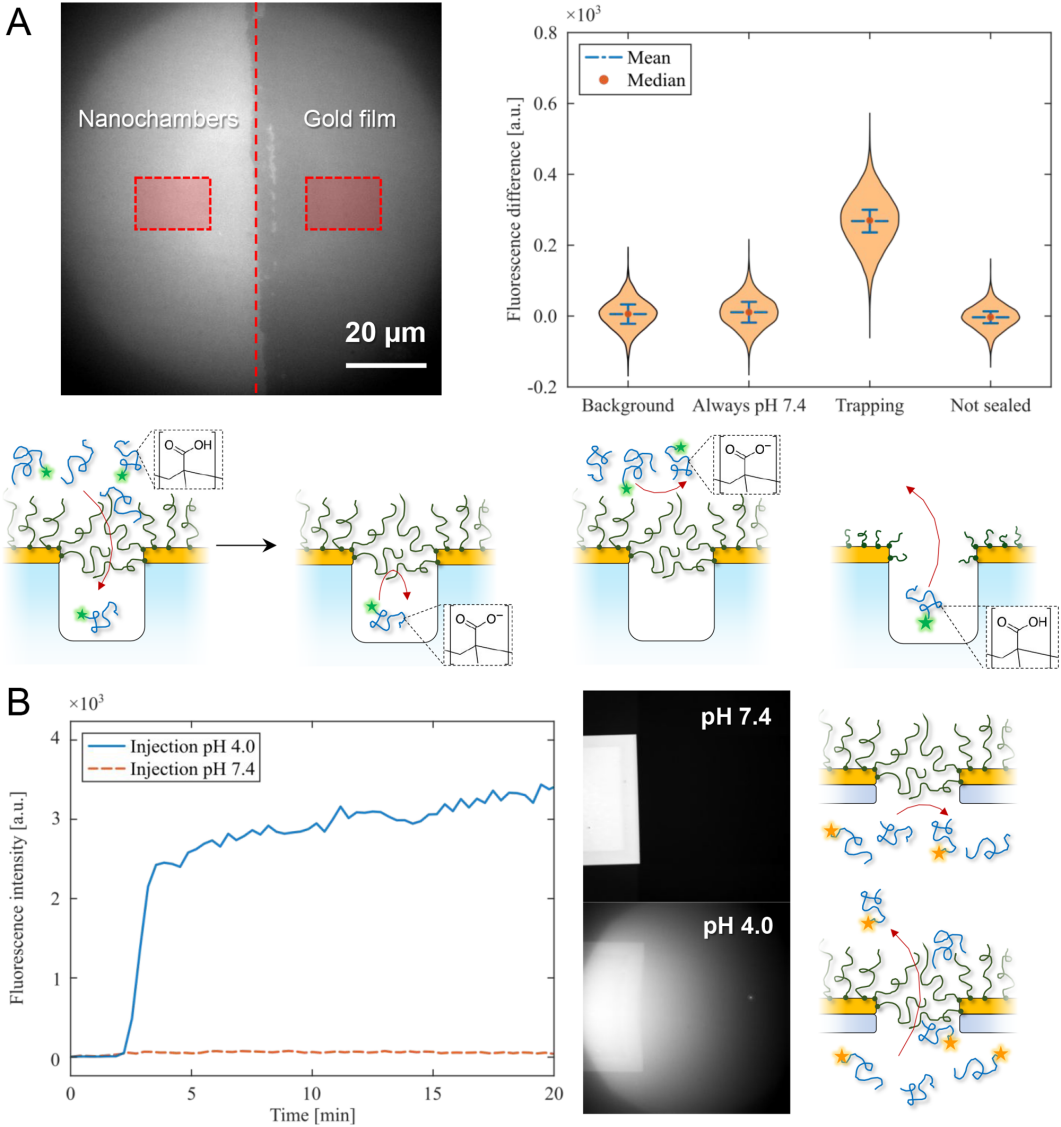
Transport of PMAA through PHEAA brushes on nanopores. (A) Fluorescence intensity differences measured from nanochambers vs planar gold with PHEAA brush. The image illustrates how intensities are collected from equal areas on the two surface regions. The border and the illumination are aligned with the center of the image. All intensities were measured after incubating with PMAA (1 mM, 50% labeled with FITC) for 30 min, rinsing away molecules in bulk, and increasing the pH from 4.0 to 7.4. The controls show (lack of) signals when the pH is not lowered during PMAA incubation and when the brush is too short to seal the opening. Violin plots illustrate intensity variations across the surface. (B) Fluorescence intensity measured vs time when injecting PMAA (0.05 mM, 25% labeled with Cy3) on the other side of a membrane with a PHEAA nanopore array at pH 4.0 vs pH 7.4. The images show one 120 μm membrane edge in fluorescence mode. The intensity values in the plots were measured 40 μm away from the membrane.

The pH-responsive transport of PMAA was further verified with the nanopore arrays (Figure 4B). Successful synthesis of PHEAA brushes on the pores was confirmed by their plasmonic resonance using extinction spectroscopy (Figure S9). Again, the targeted hydrated thickness was ∼100 nm to ensure the sealing of the pores (Figure 1). The molecules were introduced on one side of the membrane, and fluorescence was always measured at the other side, away from the membrane, to ensure that only molecules that had been transported through the pores contributed to the signal.^19,23,33^ Notably, no fluorescence increase was detected at all at pH 7.4, while at pH 4.0, the signal steadily increased. Since the receiving side of the membrane had a fairly large volume (50 μL), it can be assumed that the concentration inside remained negligible for a long time, and the transport should be in a quasi steady-state. We derived the following expression (see Figure S10 and related discussion) for the flux of PMAA molecules through the membrane with pores:

2 $$J=\frac{k_{\mathrm{on}}C_{0}\Gamma_{\max}}{1+\frac{1}{\beta }+\frac{C_{0}}{K_{D}}}$$

Here, β is the area fraction of the membrane containing pores (20% in our structures). Using the SPR data, we estimated Γ_max_ to ∼4 nm^–2^ for a 100 nm hydrated PHEAA brush (assuming binding capacity scales linearly with brush thickness). The other parameters in eq 2 are also known from the SPR experiments (table in Figure 3E). Inserting values reveals a high transport efficiency, such as 122 net translocations per pore and second at *C*_0_ = 100 μM or 1099 at *C*_0_ = 1 mM (considering only the weak binders). The kinetic parameters are valid at these concentrations (Figure 3D). For comparison, a real NPC can translocate up to ∼1000 karyopherins per second,^39^ although it is physically smaller. Hence, we conclude that our artificial NPC has a competitive transport rate in comparison with the biological system, while keeping in mind that the values are only indirectly determined through a model.

We further investigated whether the shuttle-cargo transport mechanism was compatible with other chemical conjugation schemes. Alkyne-terminated PMAA with different molecular weights was synthesized by ATRP in the bulk. Using SPR and QCMD, we confirmed that the homebrewed PMAA also behaved similarly to the commercially obtained batches with respect to binding kinetics and pH dependence (Figure S11) (However, it should be kept in mind that if the molecular weight of PMAA reaches the range of tens of kg/mol, this will increase the critical pH since longer chains enable a higher number of bonds.^18^ ). Click chemistry based on alkyne–azide cycloaddition^40^ was used to attach a dye to the PMAA as proof of concept (Figure 5A). The construct could be transported through nanopores in a pH-responsive manner, just like the other PMAA (Figure 5B). This compatibility with click chemistry opens up further possibilities for using conjugated PMAA chains to enable selective transport and trapping.

**Figure 5:**
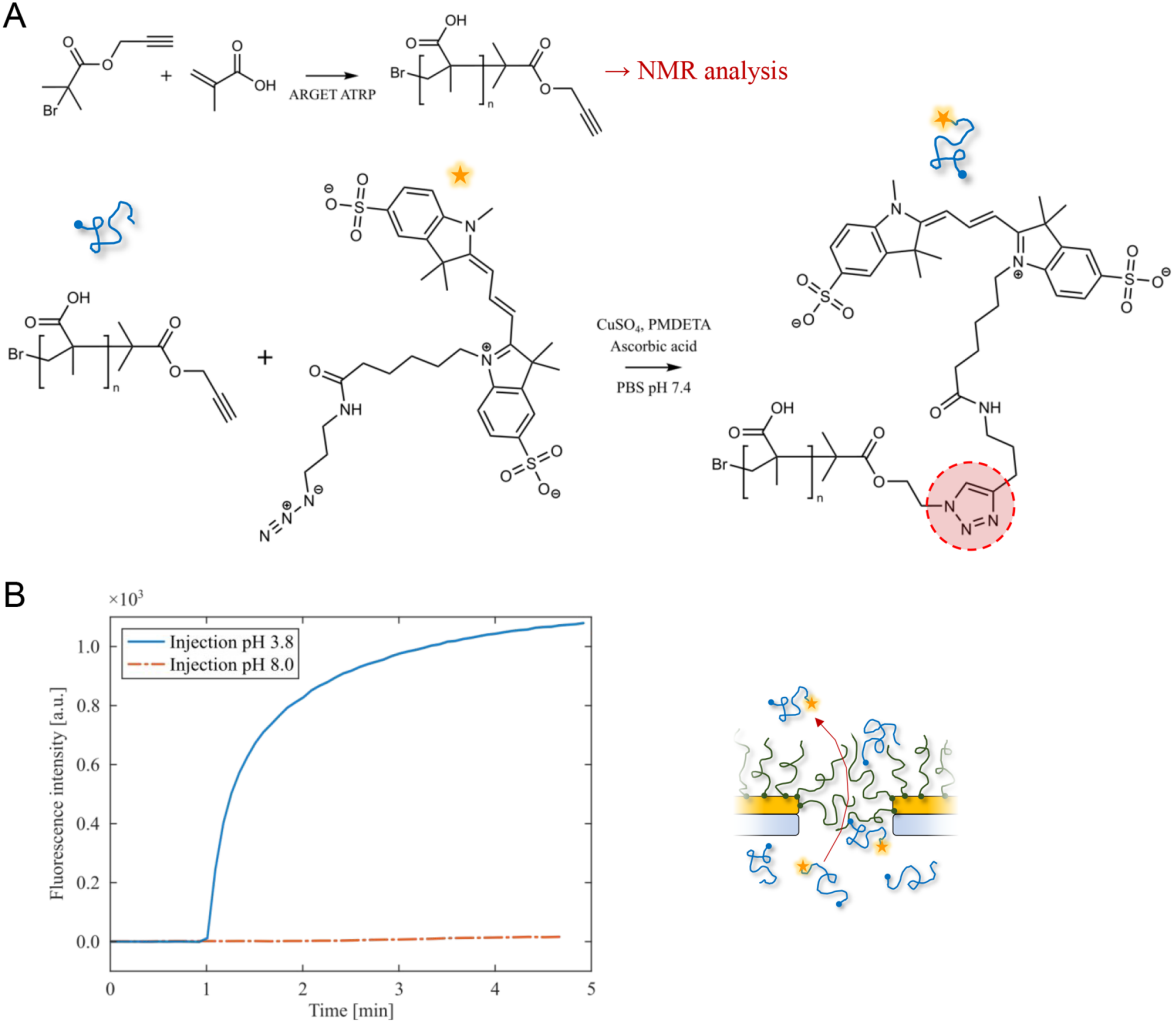
Compatibility with click chemistry. (A) Scheme of ATRP and alkyne–azide cycloaddition for specific attachment to the PMAA shuttle (here for sulfo-Cyanine3 azide). (B) Transport through nanopores using 7.8 kg/mol PMAA (1 g/L) clicked to dye (labeled fraction ∼10%). The experiments were performed similarly to those in Figure 4B.

While the results above show that the brush barrier blocks macromolecules such as proteins and that efficient pH-sensitive transport of the PMAA shuttle is possible, the “cargo” was only fluorophores. In their free form, such dyes are small enough to freely diffuse through the PHEAA barrier (Figure 2D). To fully mimic shuttle-cargo transport as performed by the NPC, the cargo should be so large that it cannot pass through the pores without facilitated transport by the shuttle. To investigate this aspect, we conjugated single-stranded DNA as cargo to the PMAA shuttles (scheme in Figure S12). First, we tested if free DNA strands could pass through the PHEAA barrier using nanopores (Figure 6A). A slow but significant transport was observed for 5 nucleotides (total weight ∼ 2.3 kg/mol with dye), but not for 10 or 15 nucleotides. Furthermore, when free PMAA (not conjugated to DNA) was also introduced and the pH further reduced to 3.5, the 10-nucleotide DNA could pass through the pores, confirming that the brush does indeed collapse (opened pores) when the affinity is too high.

**Figure 6:**
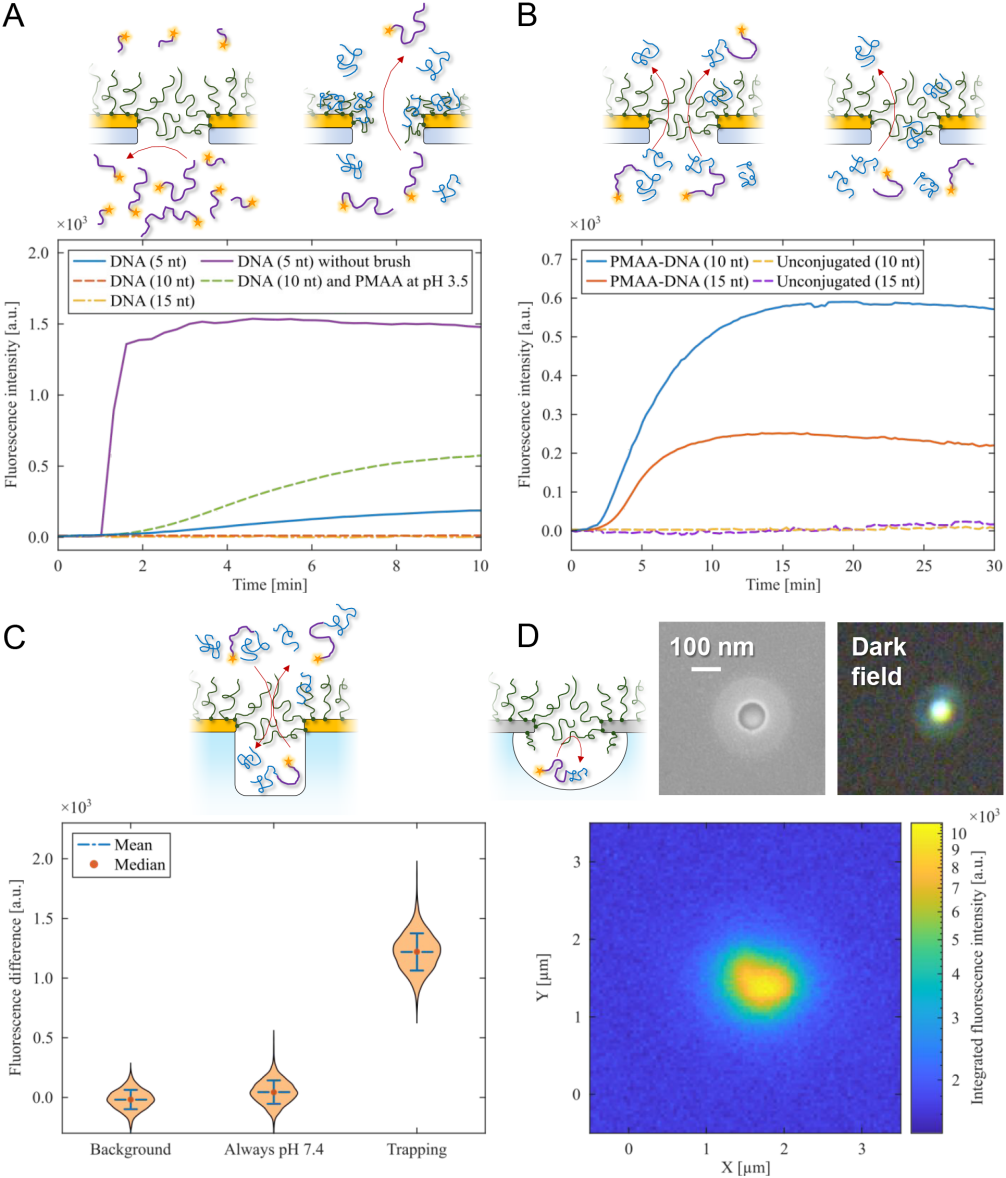
Shuttle-cargo transport mimicking nucleic acid export through the NPC. (A) Diffusion of single-stranded DNA of different lengths (5, 10, or 15 nucleotides) through PHEAA-modified nanopores. All strands were introduced at 5 μM and labeled with Cy3. In the presence of 0.5 mM PMAA at sufficiently low pH, the DNA can pass through. (B) Shuttle-cargo transport without any detectable leakage of cargo (DNA), even in the presence of free shuttles (PMAA). All concentrations are kept identical (500 μM PMAA and 10 μM DNA), but in the control experiments, the DNA is not conjugated to the PMAA. Note that all DNA strands are labeled, while PMAA is here not labeled at all. (C) Verifying trapping of a PMAA-DNA conjugate (10 nucleotides) inside nanochambers after equilibrium with 500 μM PMAA where 2% of the chains had conjugated DNA strands. The fluorescence intensity from a control region without nanochambers is subtracted (as in Figure 4A). (D) Confocal image (integrated in *z*) of DNA conjugates securely trapped in a single nanochamber with volume of a few aL. Images of such a Pd nanochamber are also shown. (The schematic shows one molecule for clarity, but in reality, there are probably up to ∼100 molecules trapped in the nanochamber.).

To test shuttle-cargo transport, PMAA-DNA conjugates were introduced to nanopore arrays, which showed clear fluorescent signals (Figure 6B). Importantly, these experiments were performed in the presence of 80:1 excess of free PMAA (unlabeled and unconjugated), thereby mimicking the native NPC transport, where many karyopherins pass the FG-Nup barrier in both directions without carrying any cargo. To confirm that the pores did not simply switch to an open state in the presence of PMAA (at pH 4.0), we performed control experiments with the same amounts of both species, but without any conjugation, which again led to no detectable transport. The membranes could be regenerated by washing at pH 7, and transport could be measured again in a reproducible manner (Figure S13). Furthermore, transport was gradually hindered when the pH was increased from 4.0, as expected (Figure S14). Notably, the 15-nucleotide strand has a larger molecular weight than the PMAA which carries it through the brush barrier. It was also possible to detect transport of an even larger cargo, where a second DNA strand was hybridized to the first one, albeit at lower efficiency (Figure S15). The upper limit of cargo size is expected to depend on the PMAA molecular weight, with larger shuttles enabling the transport of larger cargo. However, when using a longer PMAA, the pH optimization of interaction affinity (Figure 3B) needs to be redone.

Finally, we showed that the PMAA-DNA conjugates could be trapped inside nanochambers by raising the pH after establishing equilibrium (Figure 6C). Confocal microscopy was also used to image single nanochambers with DNA molecules trapped inside (Figure 6D). The molecules could not escape for the duration of the experiment (∼1 h), in agreement with the excellent barrier properties of PHEAA and the strict pH-control of the transport activity. We emphasize that although a temporary pH decrease is needed to get the DNA construct into the nanochambers, they eventually become trapped in a tether-free manner at physiological pH (and ionic strength).

## Conclusion

We have presented a new kind of NPC mimic based on multivalent polymer–polymer interactions. In contrast to previous studies, it exhibits very high transport selectivity, with no detectable leakage of macromolecular species. In fact, at least for passive diffusion, the selectivity of our artificial construct is most likely higher than that of the real NPC, which actually does allow larger proteins to pass over longer time scales.^41,42^ Furthermore, we have shown that “cargo” molecules do not leak through the pores even in the presence of the “shuttle” molecules, i.e., the pores are not opened/closed by the interactions that occur. This critical feature was realized by carefully tuning experimental parameters, in particular, pH. We emphasize that key to success is to find conditions where transporters can move through the intrinsically disordered polymer brush barrier by multivalent interactions without influencing its morphology. The heteromolecular polymer interactions are probably due to a combination of hydrophobic groups and hydrogen bonds. Regardless, we postulate that any kind of multivalent weak interactions, where each bond is a few *k*_B_ *T* in strength, can achieve the same result. Most likely, the NPC is similarly fine-tuned in structure so that equilibrated binding with karyopherins does not alter the morphology of the FG-Nup barrier too much, or at least does not collapse it. The transport capacity was found to be at least ∼1000 PMAA molecules per second in our artificial pores based on a model with binding/release kinetics.

Taking advantage of the pH-responsive transport mechanism, our system can also be used for various applications. This may involve selective ultrathin membranes with transport “on demand”. The compatibility with click chemistry enables further possibilities for the separation of selected targets tagged to the transport shuttles. In this first study based on heteromolecular polymer interactions, we show that it is possible to trap molecules inside nanochambers by switching the polymer brush barrier to a repelling state after letting targets diffuse into the chambers. This shows a concept for trapping molecules where the brush no longer needs to be responsive in terms of morphology changes.^23^ The molecules become trapped in a tether-free manner at physiological conditions, without any force acting upon them. This feature may be extremely useful in single-molecule studies, where there is a great need to extend the observation time to enable observation of biomolecular reactions and interactions.^43^ Another interesting next step in this line of research is to also test transport of cargo proteins. However, this will require extra considerations since the conjugation chemistry becomes less straightforward and since PMAA interacts with proteins at low pH.^44^ Protein transport may be easier to achieve with other polymer–polymer interactions, such as ion-pairing in zwitterionic polymers.^13^

## Experimental Section

### Chemicals

All chemicals used were purchased from Sigma–Aldrich unless stated otherwise. Water used was ASTM research-grade Type 1 ultrafiltered water (MQ, 18.2 MΩcm). Hydrogen peroxide (H_2_O_2_, 30%) and ammonium hydroxide (NH_4_OH, 28–30% in water) were from ACROS chemicals or Thermo-Fischer Scientific. The initiator for ATRP was 11-mercaptoundecyl 2-bromo-2-methylpropanoate from Chemtronica. The chemicals used for polymer synthesis were *N*-hydroxyethyl acrylamide (HEAA), tris­(2-dimethylaminoethyl)­amine (Me_6_TREN), CuBr_2_ and l-ascorbic acid. PMAA with α-amino-termination was purchased from Polymer Source Inc. The chemicals used for the synthesis of PMAA with alkyne terminal were propargyl alcohol, triethylamine, α-bromoisobutyryl bromide, MgSO_4_, CuCl_2_, tris­(2-pyridylmethyl)­amine (TPMA), and methacrylic acid. Fluorescent dyes used were sulfo-Cyanine3 NHS ester, sulfo-Cyanine3 azide, AF430 NHS ester (all from Lumiprobe) and NHS-Fluorescein (purchased from Thermo-Fischer Scientific). FITC-conjugated albumin from bovine serum (BSA) was purchased from Thermo-Fischer Scientific. Cy3-labeled (5′) single-stranded DNA were purchased from Integrated DNA Technologies. Sequences were TGGAA, TGGACATCAA, and TGGACATCAGAAATA. The (3′) ends had disulfides. Chemicals used for the conjugation of DNA to PMAA were sulfosuccinimidyl 4-(*N*-maleimidomethyl)­cyclohexane-1-carboxylate (sulfo-SMCC, Thermo-Fischer Scientific), tris­(2-carboxyethyl)­phosphine (TCEP) and 1,17-diazido-3,6,9,12,15-pentaoxaheptadecane. Chemicals used for the alkyne–azide click-chemistry were CuSO_4_, N,N,N’,N”,N”-pentamethyldiethylenetriamine (PMDETA) and l-ascorbic acid. Unless stated otherwise, the buffer used in all measurements was phosphate-buffered saline (PBS) containing 10 mM monosodium and disodium phosphate, 137 mM NaCl, and 2.7 mM KCl. The pH was adjusted with 1 M HCl or 1 M NaOH and was controlled within ± 0.05 units.

### Nanostructure Fabrication

Nanopores in gold and silicon nitride were fabricated using electron beam lithography, as described previously.^38^ In brief, a negative resist was used to create pillars, followed by the deposition of metal and a protective Al_2_O_3_ film, after which the pores were created by plasma etching. The silicon nitride thickness was either 20 or 50 nm, while the gold was always 30 nm. The membranes were either approximately 30 × 30 μm^2^ (more stable) or 120 × 120 μm^2^ (higher throughput). Fabrication of nanochambers with Au or Pd for trapping has been described previously.^23^ The surface density of nanochambers was either as in Figure 1 or strongly reduced for imaging of single chambers.

### Surface Plasmon Resonance

Glass substrates used with the SPR were purchased from BioNavis. All SPR sensor chips were manufactured in-house by depositing Cr (2 nm) followed by Au (50 nm). Measurements were performed on a SPR Navi 220A instrument (BioNavis). The flow rate of the buffer used was 25 μL/min and all measurements were done at 25 °C. Analysis of SPR spectra by Fresnel modeling to determine dry and hydrated thickness has been described in previous work.^36^ The refractive index of PHEAA was set to 1.464 (670 nm), 1.457 (785 nm), and 1.449 (980 nm) based on spectroscopic ellipsometry (Figure S16). The polymer density was set to 1.05 g/cm^3^. Sensorgrams are shown for the 785 nm wavelength unless otherwise stated.

### Quartz Crystal Microbalance

Crystals were purchased from QuartzPro. Measurements were performed with a Q-Sense E4 instrument (Biolin Scientific) equipped with a peristaltic pump (Ismatec). The flow rate of buffer was 100 μL/min, and all measurements were done at 25 °C. Data are shown for the fifth overtone unless otherwise stated.

### Surface Preparation

SPR and QCMD surfaces were cleaned with an RCA1 wash: 1:1:5 volume ratio NH_4_OH, H_2_O_2_ and MQ water for 20 min at 75 °C, rinsed with MQ water and ethanol, dried under a flow of N_2_ and then cleaned with UV/O_3_ (placed under a 90 W mercury vapor lamp for 10 min). Nanopore membranes and nanochambers with gold surfaces were etched in NaOH (0.01 M) for 45 min to remove the protective Al_2_O_3_ layer^38^ and then cleaned with UV/O_3_. Nanochambers with Pd surfaces were sonicated in acetone for 1 min and in ethanol for 1 min. The initiator layer was formed with a 2.0 mM ethanolic solution overnight (at least 18 h). After self-assembly, samples (except nanopore membranes) were sonicated (35 kHz) in ethanol for 1 min and dried with N_2_.

### Surface-Initiated Polymerization

ATRP with activators regenerated by electron transfer (ARGET) was used to synthesize PHEAA brushes. CuBr_2_ (2.5 mg, 0.01 mmol), Me_6_TREN (30 μL, 0.12 mmol), and HEAA (3.33 g, 28.9 mmol) were added to methanol (30 mL) and water (10 mL). The solution was deoxygenated with N_2_ for 1 h. The reaction solution was then transferred via cannula into a screw-top jar (with a rubber septa lid) containing initiator-prepared surfaces. The reaction was started by the addition of l-ascorbic acid (0.020 g, 0.11 mmol) in water (0.5 mL) and was quenched by immersing the surfaces in ethanol, after which the surfaces were rinsed in acetone and ethanol and dried under flow of N_2_. Chemical characterization was performed by FTIR spectroscopy (Figure S17) using a PerkinElmer in attenuated total reflection mode. All QCMD sensors, nanopores, and nanochambers were polymerized in parallel with an SPR sensor to obtain the thickness of the film.

### Synthesis of PMAA with Alkyne Terminal Group

The initiator, propargyl 2-bromoisobutyrate, was prepared as described by Doran et al.^45^ Cu(0)-mediated ATRP was performed with CuCl_2_ (1.8 mg, 0.01 mmol), TPMA (15.8 mg, 0.05 mmol), methacrylic acid (1.19 mL, 14.0 mmol), and the initiator (0.02 mL, 0.14 mmol) mixed in a 5:4:1 (5 mL) solution of dimethyl sulfoxide, MQ water, and 1 M hydrochloric acid. The reaction mixture was deoxygenated by nitrogen purging for 30 min. A copper wire was sand-papered and immersed in concentrated hydrochloric acid to eliminate oxide layers. The reaction was initiated by transferring the solution to a round-bottom flask containing the clean copper wire, which acted as a catalyst. The reaction was left to stir for 5 h while maintaining an inert atmosphere under nitrogen. The synthesized PMAA was purified with a 3.5 kg/mol cutoff dialysis membrane (SnakeSkin, VWR) for 3 days with regular water exchanges. The cation exchanger Dowex Marathon MSC was added to the dialysis bag to capture any residual copper ions. Following dialysis, the solution was gravimetrically filtered through paper and subsequently freeze-dried under vacuum for 24 h before storage in a freezer. NMR (400 MHz Varian spectrometer) was used to verify the product and estimate molecular weight.^46^ In brief, we integrated the vinyl proton (δ) at ∼5.8 ppm (monomer) and the α-methyl group (α) at ∼0.8 ppm (polymer) (Figure S18). The vinyl proton was integrated and normalized to 1.0 (H × 1), with a relative signal of 1.0. The α-methyl group was integrated to 29.4 (H × 3), with a relative signal of 9.8. Overall proton signal from both monomer and polymer was 10.8, which gave a monomer conversion of 90.7%. The target *M* was 8.6 kg/mol based on the ratio of initiator and monomer, and the actual *M* was calculated to be 7.8 kg/mol (∼91 units) based on the monomer conversion.

### Alkyne–azide Click Chemistry

Alkyne-terminated PMAA (11.7 mg, 1.5 μmol) and l-ascorbic acid (0.5 mg, 3.0 μmol) were mixed with PBS (1.75 mL) at pH 7.4. Azide-terminated sulfo-Cyanine3 (0.12 mg, 0.17 μmol) was mixed with water (0.15 mL) and added to the PMAA solution. CuSO_4_ (5.5 mg, 0.02 mmol) and PMDETA (7 μL, 0.03 mmol) were mixed with MQ water (10 mL) in a separate flask. Both solutions were deoxygenated by nitrogen purging, and the reaction was initiated by injecting 0.1 mL of the CuSO_4_/PMDETA mixture into the solution with PMAA and dye, and was left to stir overnight. The conjugated PMAA was purified with a 3.5 kg/mol cutoff dialysis membrane for 3 days with regular water exchanges. The cation exchanger Dowex Marathon MSC was added to the dialysis bag to capture any residual copper ions. Following dialysis, the solution was freeze-dried under vacuum for 24 h before storage in a freezer.

### Dye Conjugation

α-Amino-terminated PMAA (10.0 mg, 2.0 μmol) was mixed with 2.5 mL HEPES buffer at pH 8.3. An NHS-containing fluorescent dye (AF430 or Cy3, 2.0 μmol) was mixed with 2.5 mL HEPES buffer at pH 8.3. The reaction was initiated by mixing the two solutions and was left to stir overnight. The conjugated PMAA was purified with a 3.5 kg/mol cutoff dialysis membrane for 3 days with regular water exchanges. After dialysis, it was immediately used or stored in a freezer.

### DNA Conjugation

α-Amino-terminated PMAA (5.0 mg, 1.0 μmol) and sulfo-SMCC (1.7 mg, 5.0 μmol) were mixed with 2.0 mL PBS at pH 7.0 and reacted for 60 min to generate maleimide-terminated PMAA. Excess sulfo-SMCC was removed by dialysis in water at pH 5.0 with a 3.5 kg/mol cutoff dialysis membrane over 6 h with two water exchanges. To generate thiol groups from the disulfides, DNA (0.1 μmol) and TCEP (0.3 mg, 1.0 μmol) were mixed in 2.0 mL PBS at pH 7.2 and reacted for 60 min while deoxygenating by N_2_ purging. 1,17-Diazido-3,6,9,12,15-pentaoxaheptadecane (3 μL, 10.0 μmol) was mixed with 0.1 mL PBS at pH 7.0 and added to the DNA/TCEP solution for 60 min while continuously deoxygenating to quench any remaining TCEP.^47^ PBS salts were added to the maleimide-terminated PMAA and the pH was adjusted to 7.0. The reaction was initiated by mixing the two solutions which were then left overnight. The conjugated PMAA was purified with a 7 kg/mol cutoff dialysis membrane for 3 days with regular water exchanges. After dialysis, the product was immediately used or stored in a freezer.

### Fluorescence Microscopy

Measurements were performed with an inverted Axio Observer optical microscope equipped with an Andor IXon Life CCD, a Colibri 7 LED light source, and a 63× objective (water immersion, NA = 0.9, WD = 2.4 mm) in epi-mode. The same buffer conditions used for the sample were used for the immersion liquid of the objective. A custom-made flow cell (Figure S19A, inner volume ∼2 μL) equipped with a NE-1000 syringe pump (New Era Pump Systems) and a manual injection valve (Genetec) was used for flow control and for purging air bubbles. The volume of the liquid droplet connecting the sample and the objective was 50 μL. For detection of FITC, a beam splitter that transmits light above 499 nm was used with an emission filter transmitting between 500 and 550 nm. Excitation was achieved with an LED emitting between 450 and 488 nm. For detection of AF430, a beam splitter that transmits light above 499 nm was used with an emission filter transmitting between 500 and 550 nm. Excitation was achieved with an LED emitting between 401 and 445 nm. For detection of Cy3, a beam splitter that transmits light above 555 nm was used with an emission filter transmitting between 565 and 605 nm. Excitation was achieved with an LED emitting between 489 and 533 nm. All images shown in the same figure were acquired using the same parameters for illumination intensity and exposure time. Images acquired were converted to TIFF and extracted for data analysis in MatLab. Violin plots were made using the default kernel density estimation in MatLab.

### Confocal Microscopy

Measurements were performed with a Nikon Ti-E A1+ confocal laser scanning microscope equipped with a 60× objective (oil immersion, NA = 1.4, WD = 0.14 mm). A custom-made flow cell (Figure S19B, inner volume ∼100 μL) equipped with a NE-1000 syringe pump and a manual injection valve (Genetec) was used for flow control and for purging air bubbles. The oil immersion objective was in direct contact with the glass substrate for FRAP analysis and for nanochamber experiments. For detection of AF430, a beam splitter that transmits light above 488 nm was used with an emission filter transmitting between 500 and 550 nm. Excitation was achieved with a laser emitting at 409.2 nm, with the pinhole set to 1.2 AU. For detection of Cy3, a beam splitter that transmits light above 488 nm was used with an emission filter transmitting between 552 and 618 nm. Excitation was achieved with a laser emitting at 485.0 nm, with the pinhole set to 1.2 AU. The detector was a photomultiplier tube. The frame rate was 0.5 Hz, and the resolution was 1024 × 1024 pixels. Bleaching during the FRAP measurements were done with repeated cycles of high-intensity exposure with the 409.2 nm laser. The FRAP recovery curves were analyzed following the protocol in the software (NIS-Elements AR).

## Supplementary Material



## Notes

PEG injection in SPR, antifouling sensorgrams, temperature dependence of PMAA binding, QCMD long measurement, fits to dissociation curves, quantification of the SPR signal, PMAA binding to initiator, binding of PMAA with amine terminal, microscale extinction spectra, theory of transport rate, binding of PMAA with alkyne terminal, DNA conjugation scheme, additional DNA transport data, DNA transport at other pH, transport of larger DNA, spectroscopic ellipsometry, FTIR spectrum, NMR spectra, flow cell drawings (PDF)
