# Supplementary material for: Artificial Nuclear Pore Complexes with Exceptionally Selective Shuttle-Cargo Transport
Source: ACS Nano. 2026 Jun 29;20(27):19590–602. doi: 10.1021/acsnano.6c05720 (PMC13374382; doi:10.1021/acsnano.6c05720)
Supplement: Supplementary file 1 [file nn6c05720_si_001.pdf]

Artificial Nuclear Pore Complexes with Exceptionally Selective Shuttle-Cargo Transport

Jesper Medin, Bagus Santoso, Leyla Beckerman, Radhika Vattikunta, Rebekah Hailes, John Andersson and Andreas Dahlin.

Department of Chemistry and Chemical Engineering, Chalmers University of Technology, 41296 Gothenburg, Sweden.

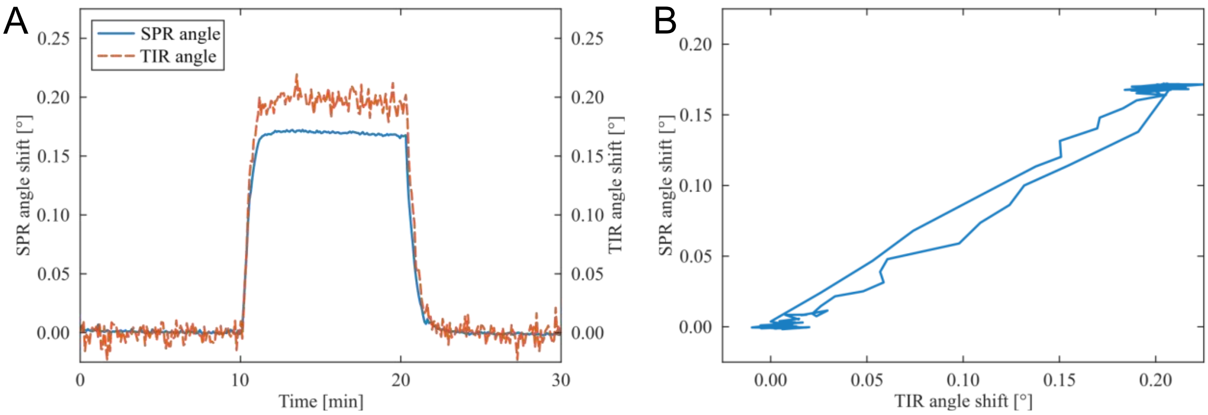

Figure S1 (A) Example injection of highly concentrated PEG solution (20 g/L) to determine wet thickness of PHEAA brushes (using 980 nm wavelength). The "exclusion height" (Figure 2B in main text) is obtained from the bulk response of the PEG as described previously.[1] It represents characteristic distance from the surface at which the PEG cannot approach further. (B) Plot of TIR angle vs SPR angle. The linearity shows that the PEG is not interacting.

[1] Ferrand-Drake del Castillo, et al. *The Journal of Physical Chemistry C* **2018**, 122 (48), 27516-27527.

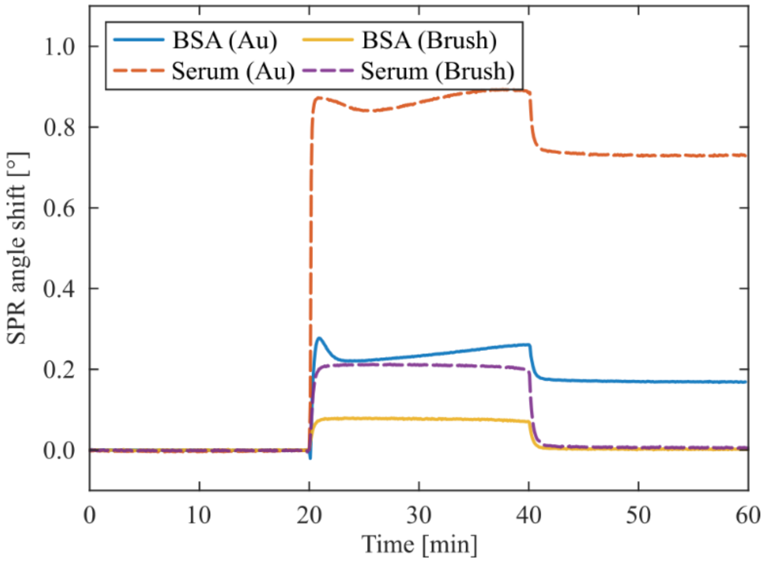

Figure S2 SPR sensorgrams (670 nm) during injections of 5 g/L BSA or 10× diluted serum in PBS. The signals during the injections are largely due to changes in bulk refractive index. The remaining signals after rinsing are used to define the fouling amount (Figure 2C in main text). The quantification into mass surface coverage was done as described previously.[2]

---

[2] Emilsson, et al. *ACS Applied Materials & Interfaces* **2015**, 7 (14), 7505-7515.

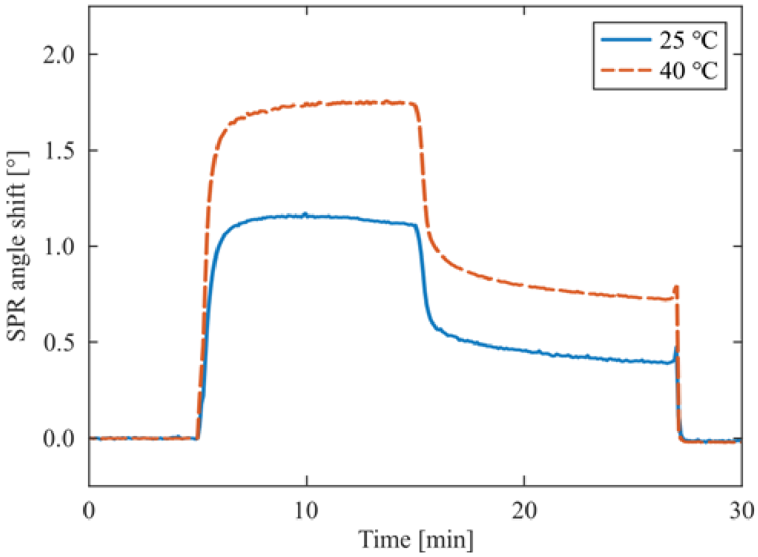

Figure S3 Temperature dependence of the interactions of PHEAA and PMAA (1 mM) at pH 4.0 followed by rinsing with PBS pH 7.4 (at 27 min). The measured affinity is higher at higher temperature, suggesting that hydrogen bonds alone cannot be responsible for the interactions.

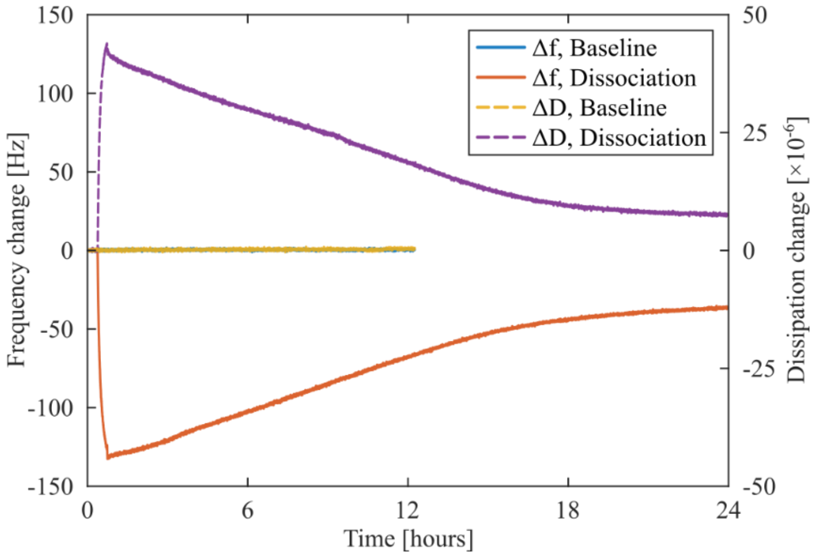

Figure S4 Dissociation of PMAA at pH 4 monitored for long time in QCMD (after binding of 1 mM in the first minutes). For comparison, a >12 h baseline is shown. The dissociation is not complete after 24 h, which is more than $1/k_{off}$ even for the strong binders, suggesting that some PMAA are even more strongly bound than what can be captured by the two-state model.

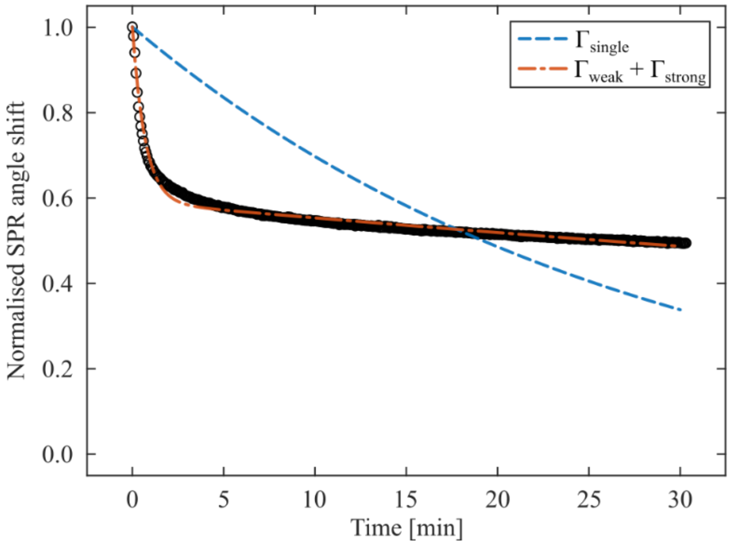

Figure S5 Example of best fits to a dissociation phase (PMAA leaving PHEAA brush at pH 4). A double exponential decay provides a much better fit than a single exponential even when taking the higher number of parameters into account. The fitting was done without a constant term, i.e. it was assumed that the curve eventually reaches zero.

Quantification of the SPR response during binding

It is well known that the SPR response is proportional to the surface coverage. To obtain a quantitative correlation we use the established field decay model:

$$\Delta\theta_{\mathrm{SPR}} = \frac{2S_0}{\delta}\int_0^{\infty} \Delta n(z)\exp\left(-\frac{2z}{\delta}\right)\mathrm{d}z$$

Here S_0 is the bulk sensitivity (98.75 degrees per refractive index unit) and δ =342 nm is the field decay length (at the 785 nm wavelength). Assuming that PMAA binds in a homogenous manner vertically throughout the brush, the integral can be written as:

$$\Delta\theta_{\mathrm{SPR}} = \frac{2S_0\Delta n}{\delta}\left\{-\frac{\delta}{2}\exp\left(-\frac{2z}{\delta}\right)\right\}_0^H = S_0\Delta n\left[1-\exp\left(-\frac{2H}{\delta}\right)\right]$$

Here H is the brush thickness, which is known. The refractive index change is related to the concentration of PMAA inside the brush and thus the surface coverage:

$$\Delta\theta_{\mathrm{SPR}} = \frac{S_0 b\Gamma}{H}\left[1-\exp\left(-\frac{2H}{\delta}\right)\right]$$

Here b is the refractive index change per concentration, which is obtained from the total internal reflection angle response in the multiparameter SPR instrument (Figure S6). This model does not rely on the common approximation that the film is much smaller than the decay length.

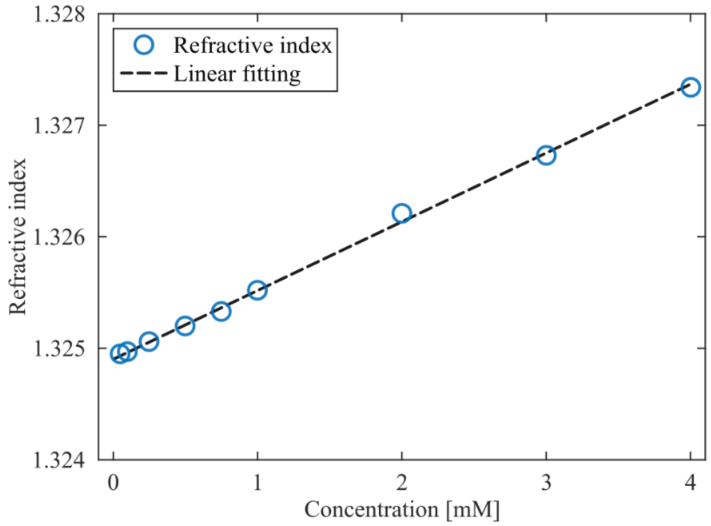

Figure S6 Determination of refractive index in bulk solutions of different PMAA concentrations using the TIR angle response. The TIR angle was converted to bulk refractive index based on its sensitivity of 74 degrees per refractive index unit. The b value (0.124 cm^3/g) is slightly lower than what we reported previously,[3] which is probably due to differences in molecular weight (which influences degree of ionization) and the wavelength used.

[3] Andersson, et al. *Langmuir* **2021**, 37 (16), 4943-4952.

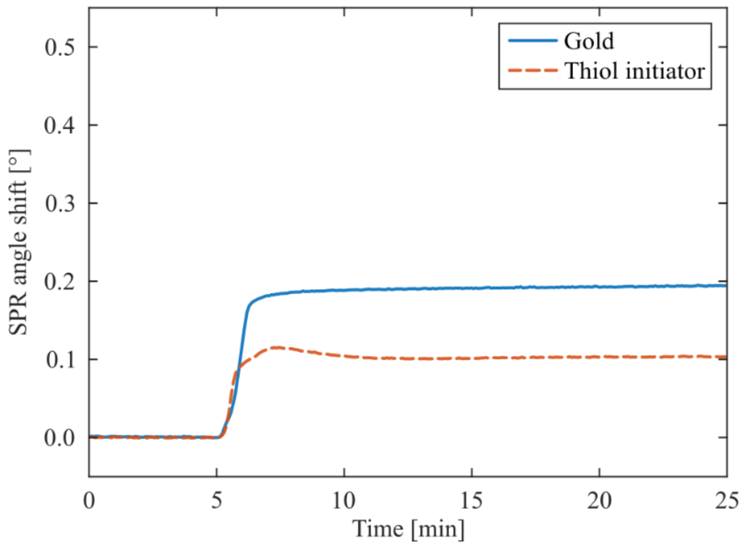

Figure S7 SPR sensorgrams during injection of PMAA (1 mM) at pH 4 to surfaces without PHEAA brushes, either bare gold or the initiator monolayer. The signals are lower than those observed for strong binding to the brushes (Figure 3C in main text), which shows that the strong binding cannot be explained simply by interactions with the underlying surface.

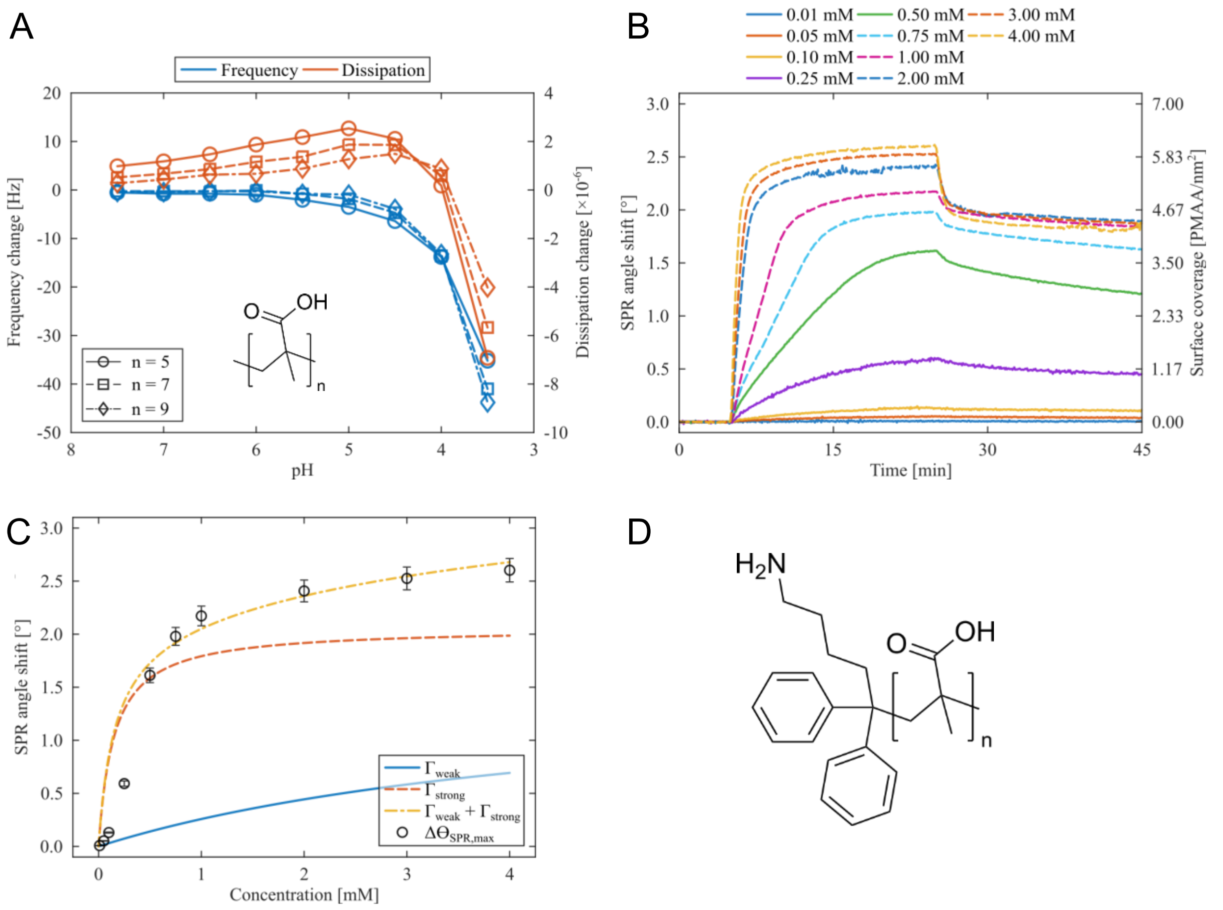

E

Binding	K_D (mM)	k_{on} ($M^{-1}s^{-1}$)	k_{off} (s^{-1})	α
1: Weak	**5.129 ± 0.864**	**4.66 ± 1.33**	**[2.39 ± 0.20] ×10^{-2}**	**0.43**
2: Strong	**0.150 ± 0.072**	**0.33 ± 0.18**	**[5.00 ± 0.76] ×10^{-5}**	**0.57**

Figure S8 Comparison of PMAA with and without an amine terminal group. (A) Critical pH analysis for ordinary PMAA (analogous to Figure 3B in main text) for PMAA with methyl termination. Note that the concentration of PMAA was lower in these measurements (200 µM). (B) Binding kinetics for amino-terminated PMAA (analogous to those in Figure 3C in main text). The exclusion height of the brush was ~110 nm. (C) Fitting to dual Langmuir model for amino-terminated PMAA (analogous to Figure 3D in main text). (D) Structure of the amino-terminated PMAA. (E) Extracted parameters for amino-terminated PMAA (to be compared with Figure 3E in main text). All these data complement those in the main text, where the QCMD results are for PMAA with amine terminal and the SPR results for ordinary PMAA. The SPR data quality and fit accuracy was slightly better for the ordinary PMAA, so the values in the table in the main text are considered most reliable. Nevertheless, the interaction kinetics are clearly similar for both polymers, which is expected since they have approximately the same molecular weight ($M \approx 5$ kg/mol).

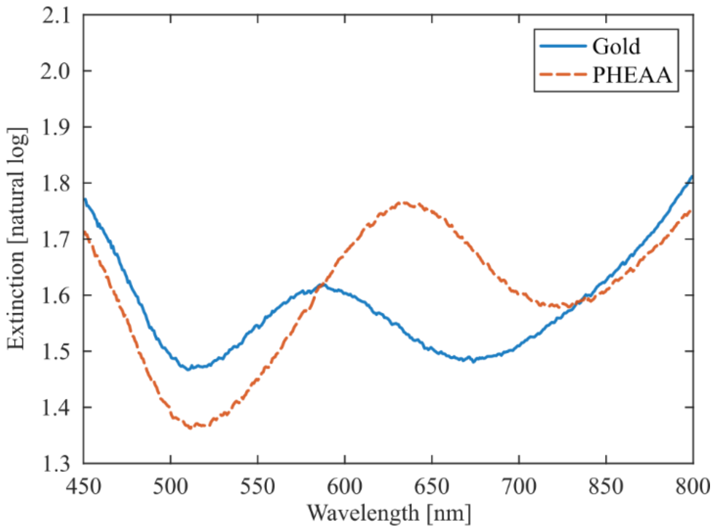

Figure S9 Microscale extinction spectra of nanopore arrays measured in air. The spectral redshift from the polymer brush is evident and similar to previous work.[4]

[4] Svirelis, et al. *Nature Communications* **2023**, 14 (1), 5131.

Theory of transport rate

We derive a rough model for the transport process in order to estimate the transport capacity, i.e. the maximal flux of PMAA molecules through the pores. The transport process can be divided into 5 steps (Figure S10):

1 Diffusion to the pore
2 Binding to the PHEAA in the pore
3 Diffusion through the brush inside the pore
4 Release from the PHEAA into the opposite reservoir
5 Diffusion away from the pore

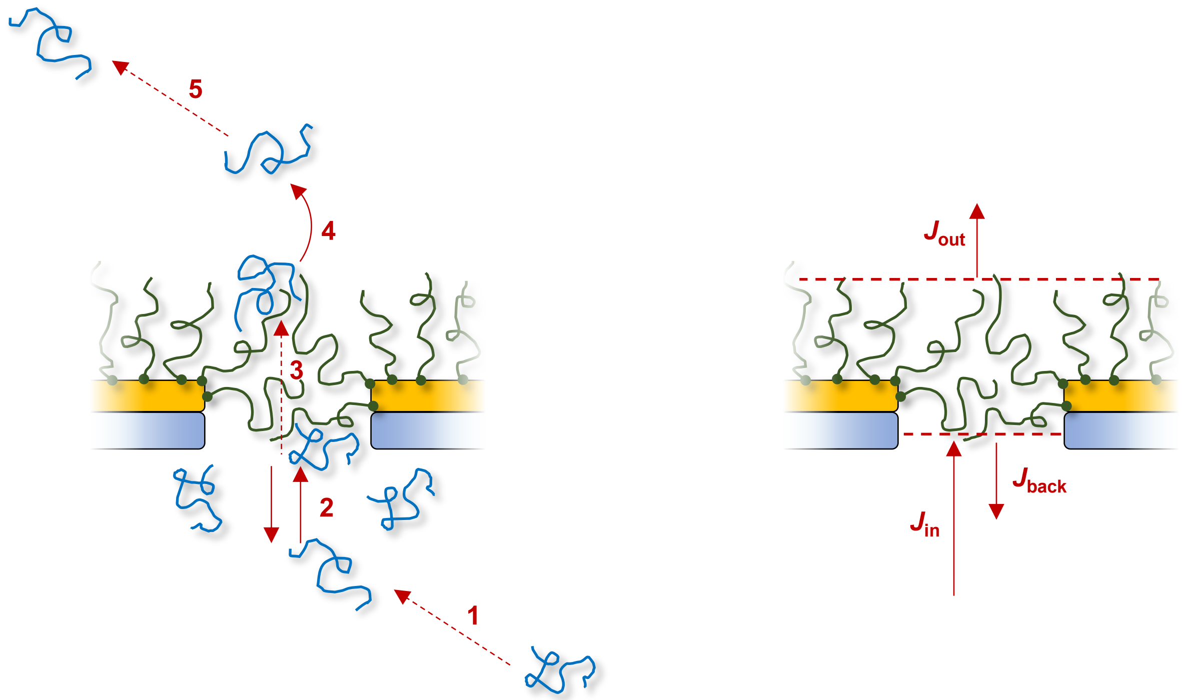

Figure S10 Model for determining transport efficiency, i.e. the flux of PMAA molecules through each pore per time unit. The process can be divided into 5 steps, but the rate limiting one is assumed to be the detachment into the receiving side.

We assume that the initial phase of the transport can be regarded as quasi steady-state with zero concentration of PMAA in the opposite reservoir. This seems reasonable since the SPR data show that equilibrium is established relatively quickly with the bulk solution on the feed side. Furthermore, the opposite reservoir is large enough to estimate the concentration as zero within for a long time.

We can also estimate whether it is reasonable that a concentration gradient is built up inside the brush based on the diffusivity of PMAA. Here we assume that the lateral diffusivity of the

strongly bound molecules (0.32 μm^2/s) can be used as a low-end estimate. Considering the thickness of the brush (100 nm albeit with some influence from the pore geometry), diffusion across the barrier should take less than 15 ms. This is much faster than the release kinetics even for the loosely bound PMAA ($1/k_{\text{off}} > 30$ s). Hence, we do not expect any concentration gradient to build up inside the brush simply because it is as extremely thin barrier. The transport kinetics are thus governed by the rate constants of binding and release. We can write the number of molecules binding and unbinding per time unit as different fluxes:

$$J_{\text{in}} = k_{\text{on}} C_0 [\Gamma_{\text{max}} - \Gamma]$$

$$J_{\text{back}} = J_{\text{out}} = k_{\text{off}} \Gamma$$

Here J_{in} and J_{back} is the binding/release towards the reservoir, while J_{out} is the flux into the empty reservoir (which we are looking for). The net transport to the opposite side can be obtained from a mass balance. In most experiments in this study, the molecules encounter the pores (the SiN_x side) before they enter the brush, which means that:

$$J_{\text{out}} A_{\text{tot}} = A_{\text{pore}} [J_{\text{in}} - J_{\text{back}}]$$

Here A_{pore} is the total area of pores and A_{tot} is the total membrane area. Rearranging and substituting we get:

$$J_{\text{out}} A_{tot} = A_{\text{pore}} \left[k_{\text{on}} C_0 \left[\Gamma_{\text{max}} - \frac{J_{\text{out}}}{k_{\text{off}}} \right] - J_{\text{out}} \right]$$

The solution for J_{out} is:

$$J_{\text{out}} = \frac{k_{\text{on}} C_0 \Gamma_{\text{max}}}{1 + \frac{A_{\text{tot}}}{A_{\text{pore}}} + \frac{k_{\text{on}} C_0}{k_{\text{off}}}}$$

The number of molecules transported per time unit through each pore is then $J_{\text{out}}/\Gamma_{\text{pore}}$ where Γ_{pore} is the surface density of pores (11 μm^{-2}).

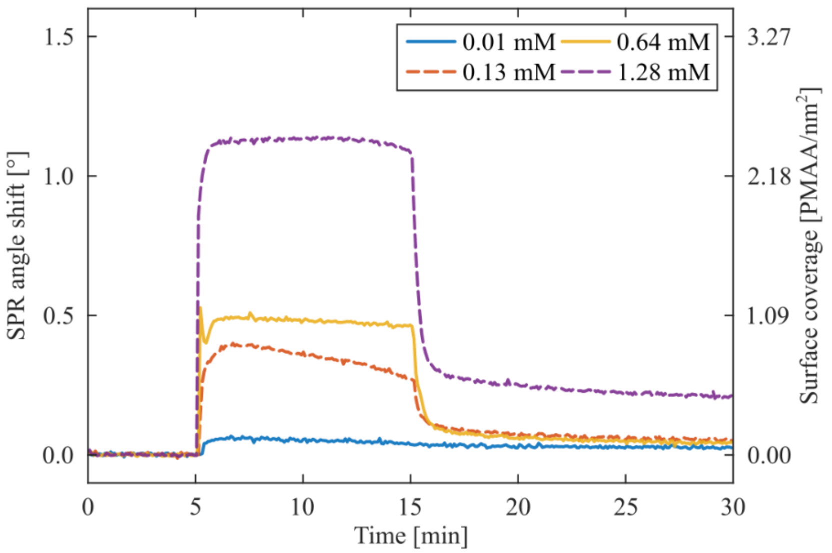

Figure S11 Example SPR sensorgrams for alkyne-terminated PMAA (7.8 kg/mol) at pH 3.8. The curves are compensated for the bulk response.[5] The exclusion height of the brush was ~90 nm. The affinity and the critical pH was found to be similar, as expected when the molecular weight is not too different.

[5] Svirelis, et al. *ACS Sensors* **2022**, 7 (4), 1175-1182.

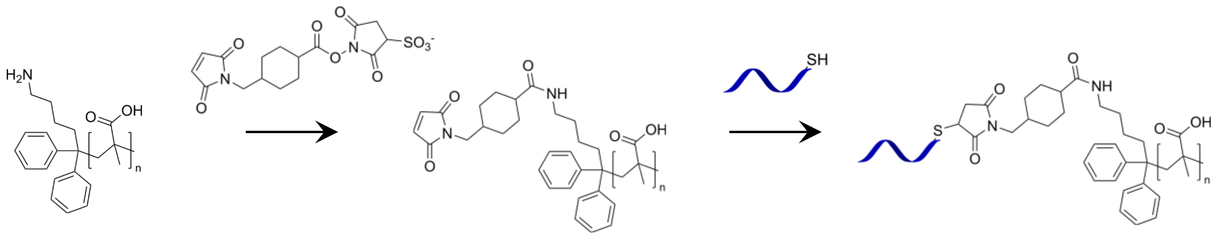

Figure S12 Scheme for conjugation of DNA to PMAA with an amine terminal group. Prior to conjugation, the DNA was treated with TCEP to reduce its disulphide group, as described in the experimental section.

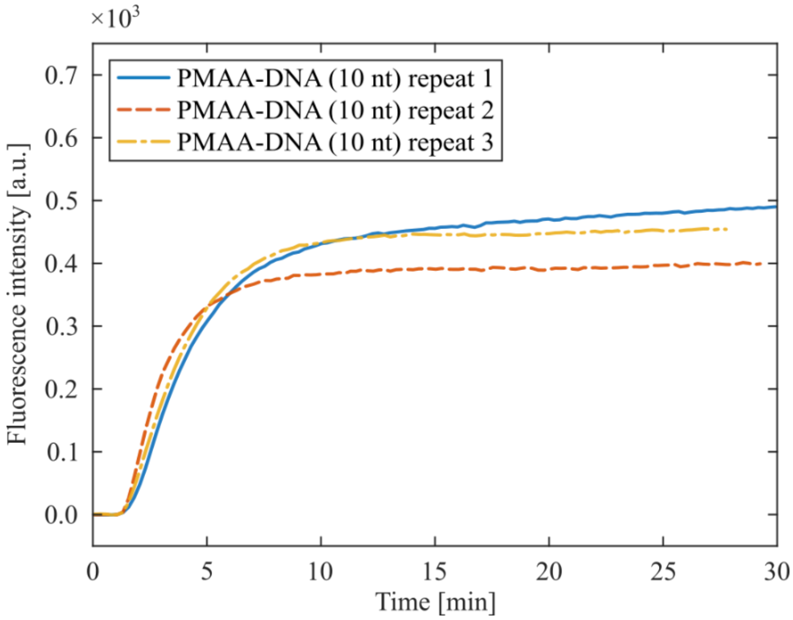

Figure S13 Typical reproducibility of shuttle-cargo transport at pH 4.0. In between each repeat the membrane is rinsed with 0.1 M NaOH and water.

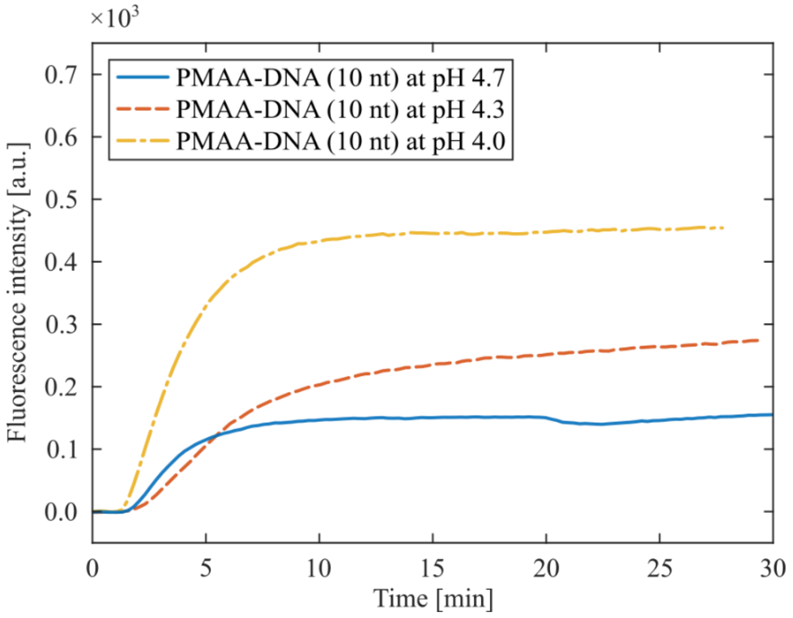

Figure S14 Examples of shuttle-cargo transport at slightly increased pH. The signal is gradually reduced when pH is increased.

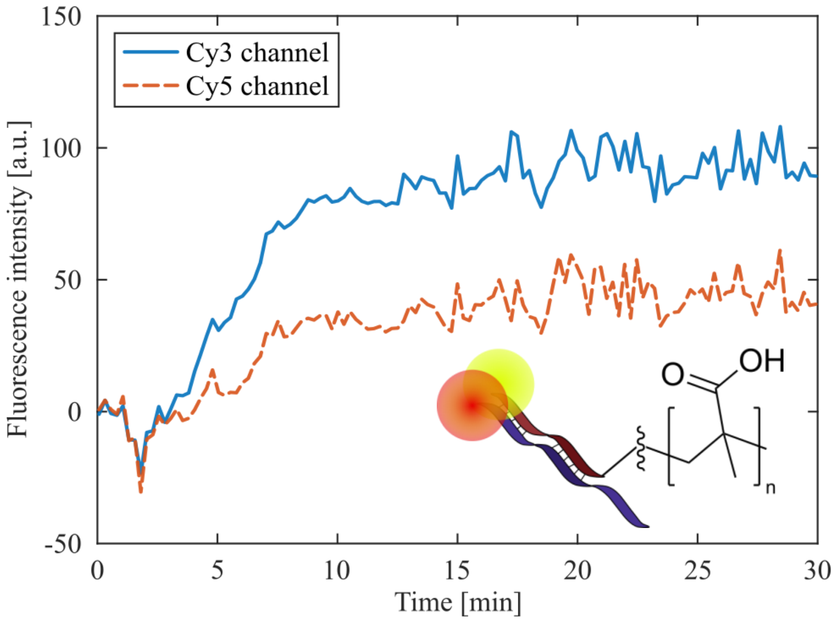

Figure S15 Shuttle-cargo transport of the 10 nt DNA to which a 15 nt strand has been hybridized (total cargo size 25 nt plus two fluorophores). The fluorescence is monitored in two different channels, one for each DNA strand, to confirm hybridization. The signals are lowered but transport clearly occurs.

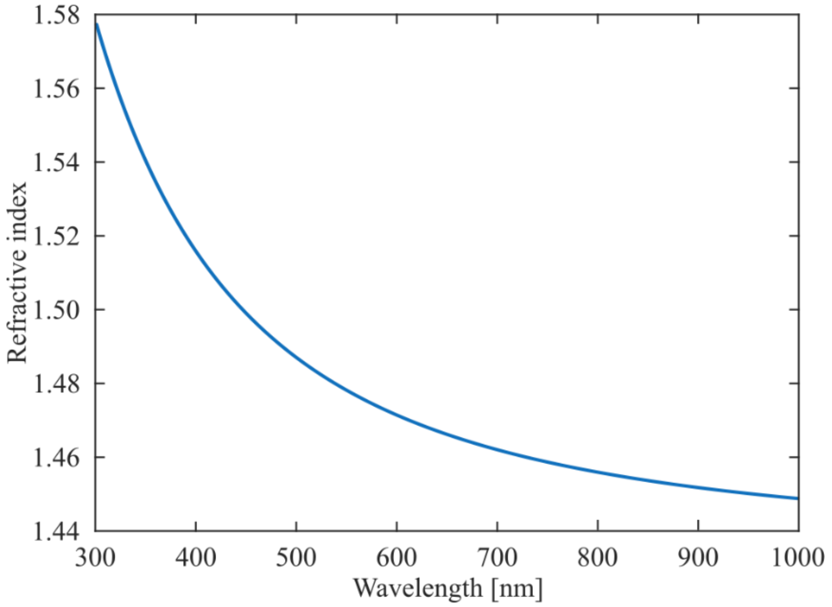

Figure S16 Spectroscopic ellipsometry to determine the refractive index of the PHEAA polymer brushes. The film was described with a Cauchy model (thickness = 32.3 nm, A = 1.436, B = 0.01270, C = 0). The extinction coefficient was negligible.

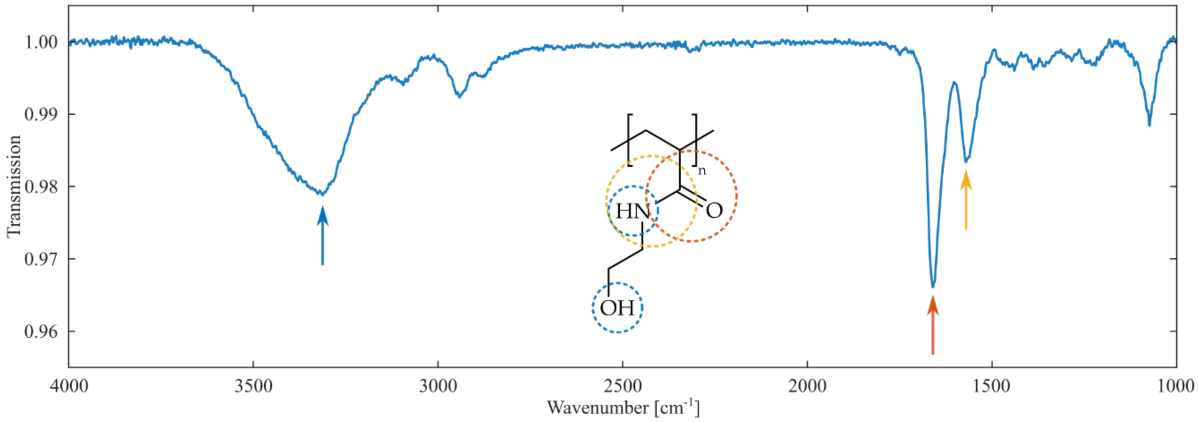

Figure S17 FTIR spectrum of PHEAA. Values are relative to a gold background spectrum. The characteristic transmission dips are identified as follows: The wide band from 3700 cm^{-1} to 3100 cm^{-1} is associated with N-H and O-H vibrations (highlighted in blue). The peak at 1650 cm^{-1} is associated with C=O stretching in the carbonyl group of an amide (highlighted in red). The peak at 1540 cm^{-1} is associated with CNH deformation of an amide (highlighted in yellow).[6]

[6] Larkin, Infrared and Raman spectroscopy: principles and spectral interpretation. Elsevier: **2017**.

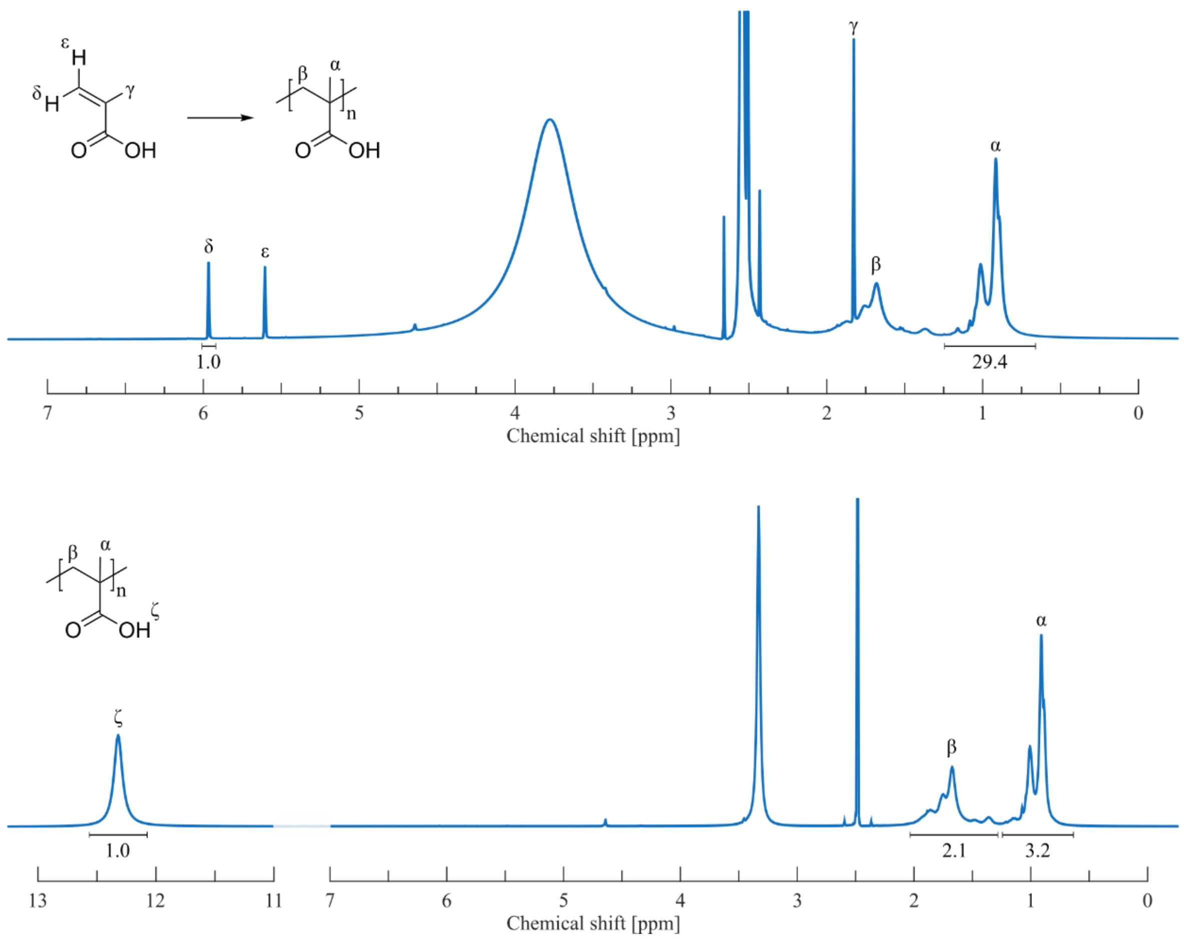

Figure S18 ^{1}H NMR spectra of crude reaction solution (upper spectra) and purified solution (lower spectra) of alkyne-terminated poly(methacrylic acid) with peak assignment highlighted with the respective chemical structure of interest. The two peaks associated with the vinyl protons of the monomer can be observed at 5.97 ppm (δ) and 5.60 ppm (ε), the peak associated with the methyl group of the monomer can be observed at 1.83 ppm (γ), the broad peak associated with the methyl group of the polymer can be observed from 0.80 ppm to 1.10 ppm (α), and the broad peak associated with the polymer backbone can be observed from 1.30 to 2.05 (β). The characteristic carboxylic acid proton, shown in the lower spectra, can be observed at 12.3 ppm (ζ). Chemical shifts are reported relative to the residual DMSO-d6 (1H, 2.50) solvent peaks according to the δ scale.

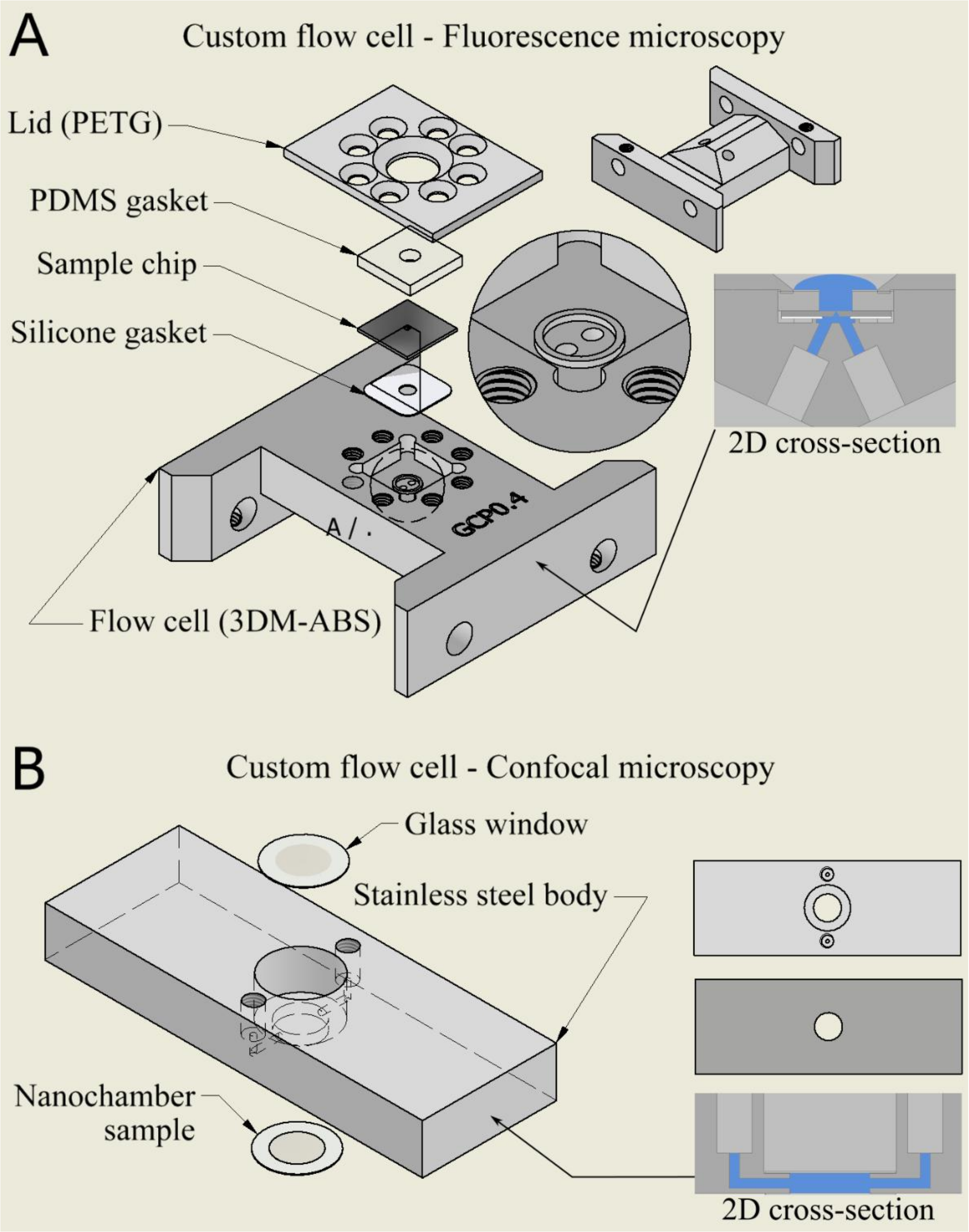

Figure S19 Drawings of flow cells used for microscopy. (A) Fluorescence imaging. (B) Confocal scanning.
